# Structural conservation versus functional divergence of maternally expressed microRNAs in the *Dlk1/Gtl2 *imprinting region

**DOI:** 10.1186/1471-2164-9-346

**Published:** 2008-07-23

**Authors:** Martin Kircher, Christoph Bock, Martina Paulsen

**Affiliations:** 1 Max-Planck-Institut für Informatik, Saarbrücken, Germany; 2 Universität des Saarlandes, Genetik/Epigenetik, Saarbrücken, Germany; 3 Max-Planck-Institut für Evolutionäre Anthropologie, Leipzig, Germany

## Abstract

**Background:**

MicroRNAs play an important functional role in post-transcriptional gene regulation. One of the largest known microRNA clusters is located within the imprinted *Dlk1/Gtl2 *region on human chromosome 14 and mouse chromosome 12. This cluster contains more than 40 microRNA genes that are expressed only from the maternal chromosome in mouse.

**Results:**

To shed light on the function of these microRNAs and possible crosstalk between microRNA-based gene regulation and genomic imprinting, we performed extensive *in silico *analyses of the microRNAs in this imprinted region and their predicted target genes.

Bioinformatic analysis reveals that these microRNAs are highly conserved in both human and mouse. Whereas the microRNA precursors at this locus mostly belong to large sequence families, the mature microRNAs sequences are highly divergent.

We developed a target gene prediction approach that combines three widely used prediction methods and achieved a sufficiently high prediction accuracy. Target gene sets predicted for individual microRNAs derived from the imprinted region show little overlap and do not differ significantly in their properties from target genes predicted for a group of randomly selected microRNAs. The target genes are enriched with long and GC-rich 3' UTR sequences and are preferentially annotated to development, regulation processes and cell communication. Furthermore, among all analyzed human and mouse genes, the predicted target genes are characterized by consistently higher expression levels in all tissues considered.

**Conclusion:**

Our results suggest a complex evolutionary history for microRNA genes in this imprinted region, including an amplification of microRNA precursors in a mammalian ancestor, and a rapid subsequent divergence of the mature sequences. This produced a broad spectrum of target genes. Further, our analyses did not uncover a functional relation between imprinted gene regulation of this microRNA-encoding region, expression patterns or functions of predicted target genes. Specifically, our results indicate that these microRNAs do not regulate a particular set of genes. We conclude that these imprinted microRNAs do not regulate a particular set of genes. Rather, they seem to stabilize expression of a variety of genes, thereby being an integral part of the genome-wide microRNA gene regulatory network.

## Background

### MicroRNA function and biogenesis

MicroRNAs are small (~20–23 nucleotides) non-coding ribonucleic acid (RNA) molecules encoded in the genomes of many eukaryotes and viruses. They bind to partially complementary sites in the messenger RNAs (mRNAs) of their target genes, thereby inducing the post-transcriptional mechanisms of gene silencing [1]. Estimates suggest that microRNAs might regulate up to 30% of all genes [2]. The presumed number of unidentified microRNAs is large [3]. Currently, about 470 microRNAs are annotated in *Homo sapiens *(human) and about 380 in *Mus musculus *(mouse) based on miRBase version 9.0. MicroRNA-encoding genes are widely distributed across a genome and occur in intergenic regions and in the introns of both protein-coding and non-coding genes. Intronic microRNAs are primarily expressed with their host gene. If not, the microRNA encoding sequences cluster at distinct genomic positions and are often coexpressed as a single polycistronic transcript within 50 kilobases [4].

In the nucleus, the RNAse enzyme Drosha and its co-factor Pasha cleave the long microRNA precursor transcript into a 70 nt to 90 nt long pre-microRNA, which has a characteristic hairpin structure. This pre-microRNA is then exported to the cytoplasm, where the enzyme Dicer processes it. The remaining microRNA duplex is incorporated into the RNA induced silencing complex (RISC). During this process the microRNA duplex is unwound and one of the RNA strands is expelled. The mature microRNA remains in the RISC and binds to partially complementary sites in the mRNAs of its target genes and prevents their translation. mRNA degradation, spatial separation or direct inhibition of the translation process may silence the transcript [1,5,6].

Like microRNA biogenesis, the target recognition process is not fully understood. The role played by RISC components also remains unclear. However, it is well established that the primary microRNA sequence is important for target recognition. Several criteria have been developed and provide the current foundation for target gene prediction. First, sequence complementarity in the seed region (bases 2–8 starting 5') of the mature microRNA is widely regarded as crucial for binding [2,7,8]. Second, 3' matches can compensate for minimal 5' pairing [9]. Third, a single mRNA sequence can contain several sites, either for one or several different microRNAs, which appears to increase silencing efficiency [10,11]. Fourth, since most microRNA binding sites are apparently located in the 3' untranslated region (3' UTR), searches for target sites are usually restricted to this area [2,9,12,13].

### Imprinting and the Dlk1/Gtl2 region

**Figure 1:**
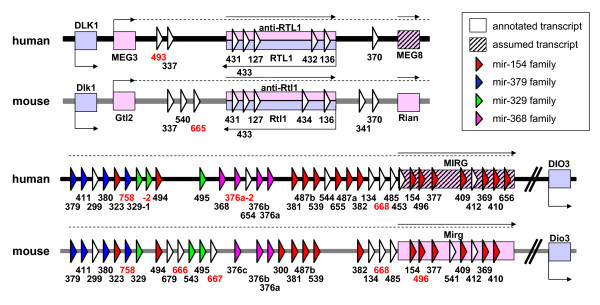
MicroRNA cluster in the *Dlk1/Gtl2 *region of human and mouse. Maternal expression of the transcripts is indicated in light magenta (*Gene-trap locus 2 (Gtl2)*/*Maternally Expressed Gene 3 (MEG3)*, *anti-Retrotransposon-like 1 (anti-Rtl1), RNA imprinted and accumulated in nucleus (Rian)*/*Maternally Expressed Gene 8 (MEG8)*, *microRNA containing gene (Mirg)*) and paternal expression in light blue (*Delta-like homolog 1 (Dlk1)*, *Retrotransposon-like 1 (Rtl1)*, *iodothyronine deiodinase 3 (Dio3)*). The figure designates by color the microRNAs (all maternally expressed) based on miRBase families with more than one member in mouse and human. Hairpin identifiers marked in red were not available in miRBase version 8.0 and were excluded from the target prediction analysis. The gene length and distances are not depicted to scale.

One of the largest microRNA clusters is on human chromosome 14q32. Its orthologous region in mouse is situated on the long arm of chromosome 12. About 10% of the microRNAs currently known in mouse and human are located in this cluster. This cluster is located within a well-known imprinted region that is characterized by parental-origin-specific mono-allelic expression of the encompassed genes (genomic imprinting is an epigenetically heritable mechanism that has been extensively reviewed [14-16]). In the *Dlk1/Gtl2 *region, six imprinted genes have been annotated; three are paternally expressed (*Dlk1/DLK1*, *Rtl1*/*RTL1*, *Dio3/DIO3*), and four are maternally expressed (*Gtl2*/*MEG3*, *anti-Rtl1*, *Rian/MEG8*, *Mirg*) [16-18]. Some of these genes have different names in mouse and human. To avoid confusion, we will only use the mouse name. While the paternally expressed genes encode proteins, the maternally expressed genes represent non-coding RNAs. Analyses of numerous microRNAs in this region revealed that the microRNAs are transcribed only from the maternal chromosome in mouse [19,20]. For their human orthologs, it is likely that these microRNAs share the murine imprinting pattern; however, this has not been experimentally validated. Pronounced transcription of the intergenic regions between *Gtl2 *and *Mirg *suggests that these microRNAs are embedded in long non-coding transcripts that may run from the *Gtl2 *promoter to as far as the 3' end of *Mirg *[20]. In the orthologous human genomic sequence, the presence of numerous expressed sequence tags (EST) suggests substantial transcription of the region downstream from *Rian*. Figure 1 depicts the 42 human microRNAs and the 42 mouse microRNAs based on information from miRBase [21] version 9.0 and the annotated transcripts for the *Dlk1/Gtl2 *region.

In both organisms the microRNA precursor sequences are organized in two groups: the first between *Gtl2 *and *Rian *[22], and the second between *Rian *and the 3' end of *Mirg *[19]. Most of the pre-microRNAs in the second cluster are arranged in tandem arrays of closely related sequences and are presumably the result of sequence duplications. Seitz et al. [19] identified the microRNAs in this second cluster and identified three different hairpin sequence families, one family with 24 members, one with six members and one with four members. These families have been incorporated into the miRBase [21] families indicated in figure 1.

Deregulation of imprinted gene expression in this region causes hypertrophy in sheep hind muscles [17], abnormalities in muscle, bone and placenta, impaired embryonic growth and death in mouse [18,23,24], skeletal malformations and various other abnormalities in human [25]. The observed phenotypes have not been mapped to specific transcripts, and the potential functional involvement of these microRNAs remains unclear. Although not extensively analyzed, strong expression of *Mirg *and the imprinted microRNAs has been observed in the mouse brain [20]. Since expression of *Gtl2 *and *Mirg *has been observed in several mouse organs, including skeletal muscle, tongue, limbs and placenta, during early stages of development [20], it appears likely that these microRNAs also appear in the tissues affected. Thus, deregulation of these imprinted microRNAs might contribute to the observed phenotypes.

The high sequence similarities observed between many of the imprinted microRNA genes suggest that the mature microRNAs may also be similar and silence the same target genes [19,26-28]. To test this, we systematically compared the pre-microRNA and mature sequences of the imprinted microRNAs on human chromosome 14 and mouse chromosome 12. As suggested by the phenotypes of the imprinting mutations and tissue-specific expression patterns, these microRNAs may silence specific subsets of genes that play a role in organ development, such as brain and muscle. Since possible target genes have been identified for only a few imprinted microRNAs, e.g. *miR-134*, *miR-376a*, *miR-370*, and the microRNAs embedded in the antisense transcript of the *Retrotransposon-like 1 (Rtl1) *gene [19,22,29-32], we established a pipeline that combines different algorithms to predict microRNA target genes computationally.

We decided to exploit the diversity of available target prediction methods by combining their results. In multiple areas of bioinformatics (e.g. protein structure prediction, protein function prediction and gene prediction) such consensus methods have achieved higher prediction accuracy and robustness than any of the underlying algorithms alone. Studying the predicted target genes of the *Dlk1/Gtl2 *microRNAs in terms of their sequence features, expression patterns and gene ontology annotations, we find that the microRNAs in the imprinted region may target a similarly broad spectrum of genes as a group of randomly selected microRNAs that are located elsewhere in the genome.

## Results

### Sequence similarities of microRNAs

We examined 31 mature microRNAs with orthologs in both human and mouse, as well as 14 uniquely human mature microRNAs and 12 microRNAs unique to mouse (see Additional file 1). We investigated the similarities among these microRNAs and to other microRNAs in each species, as well as their degree of conservation between the two species. The goal of this analysis was to understand the structural and functional similarities as well as the uniqueness of the microRNAs in the *Dlk1/Gtl2 *region more fully, and to investigate further the previous claim that this microRNA cluster emerged through tandem duplications [19,28].

**Figure 2:**
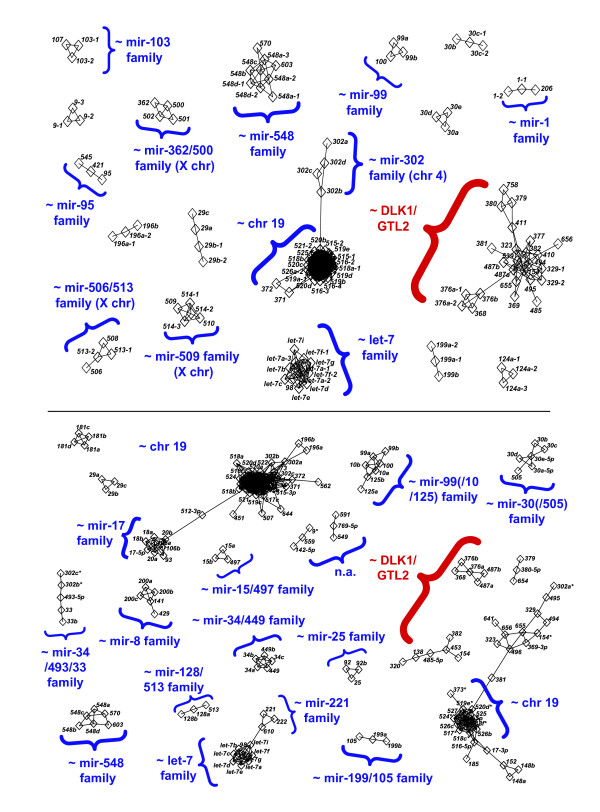
MicroRNAs in the *Dlk1/Gtl2 *region exhibit unique sequence characteristics. *Top: *Graph of human microRNA sequence similarities based on complete hairpin sequences and restricted to components with more than two nodes. *Bottom: *Graph of mature microRNA sequence similarities for human restricted to components with more than two nodes. Communities with heterogeneous microRNA identifiers are annotated based on miRBase version 9.0. The edges between nodes are shown if the similarities of the respective microRNAs are described by a similarity quotient SQ ≥ 0.75.

We combined pairwise alignment and graph analysis methods to analyze the pre-microRNA and mature sequence similarities within the *Dlk1/Gtl2 *region and also to other microRNAs at different genomic locations. To assess the sequence similarities between two pre-microRNAs or mature microRNAs, we defined the similarity quotient SQ (cutoff 0.75) as the ClustalW [33] pair alignment score divided by the minimum of the two alignment scores of each sequence. The SQ value can be regarded as a measure of similarity that is based on the shorter sequence, because conventional sequence identity would severely penalize length differences between sequences, which occur often in sequence annotation. Briefly, alignments for all pairs of microRNA sequences were calculated and graphs were generated from the resulting SQ values (with sequence identifiers as nodes and an edge between two nodes if the sequences were similar) for each species and sequence type (human: Figure 2, mouse: Additional file 2).

The pre-microRNA sequence graphs indicate that the cluster of microRNAs between *Gtl2 *and *Rian *represents an accumulation of microRNA precursor sequences that show no pronounced similarities to one another. In contrast, the second cluster between *Rian *and the 3' end of *Mirg *encompasses many pre-microRNA sequences with high similarities to one another. This confirms published data on pre-microRNA clustering, particularly in the genomic segment between *Rian *and *Mirg *[19,26-28]. Furthermore, none of the pre-microRNA sequences is similar to microRNAs encoded outside the *Dlk1/Gtl2 *region, supporting an absence of potential paralogues in different genomic regions.

The high similarity of neighboring pre-microRNA sequences in the second cluster suggests that the mature microRNAs should be similar to one another. However, comparing the graphs of the pre-microRNA and mature microRNA sequences, we noticed striking differences. In human, we see one large family of pre-microRNAs (comprising all hairpin families defined by Seitz et al. [19]) that is completely decomposed for the mature microRNA sequences. Fewer mature sequences fulfill the SQ cutoff of 0.75; therefore, smaller families and a lower connectivity within the families are observed. As shown in the alignment of pre-microRNA sequences in the largest previously defined microRNA family within the *Dlk1/Gtl2 *region [19], *mir-159 *(see Additional file 3), these differences are due to small sequence variations, shifts in the hairpin structures causing different cleavage sites and differences in the location of the mature microRNA within the hairpin (5' vs. 3' end). Overall, these differences in clustering indicate an elevated divergence of the mature microRNA sequences.

The mature microRNAs between *Gtl2 *and *Rian *are also dissimilar, which is not surprising since their precursor sequences lack similarities. As indicated by the data on pre-microRNAs, only a few mature microRNAs (six in human, two in mouse) show pairwise similarities to non-*Dlk1/Gtl2 *microRNA sequences; none exhibits more than a single link to external sequences.

Analyzing the sequence conservation between mouse and human, most mature sequences that share the same pre-microRNA identifiers show highly conserved mature sequences (average SQ of 0.986). Only the mature *miR-411 *sequences differ substantially from each other, due to a different annotation of their hairpin sequences. Based on the tandem duplication hypothesis, we also investigated whether the sequence similarity is higher for any mature sequence pairs with different pre-microRNA identifiers (all alignments are available in Additional file 4). We found only one example, *hsa-miR-376b *and *mmu-miR-376a *(SQ of 0.905 compared to 0.864 for *mmu-miR-376b*). The murine sequence *mmu-miR-376a *is one nucleotide shorter and is aligned with two instead of three mismatches. Taking the shorter sequence into account, this is no considerable difference.

Overall, our results showed high levels of sequence conservation between mouse and human microRNAs and a high diversity among the *Dlk1/Gtl2 *mature microRNAs within each species. The high diversity of mature microRNA sequences is unexpected for a functionally constrained cluster that originates from sequence duplication events, but reasonable when considering a reduced selective pressure after duplication events.

### Targets of the Dlk1/Gtl2 microRNAs

To assess whether the divergence among the mature microRNAs extends to their target genes, we predicted putative target sites in human and mouse 3' UTR sequences of protein coding genes and in repetitive elements for all microRNAs encoded in the *Dlk1/Gtl2 *region. An equal number of non-*Dlk1/Gtl2 *microRNAs was randomly selected and used as the reference for distinguishing effects specific to the microRNA cluster within this imprinted region (list of mature microRNAs: see Additional file 1). For target sequence prediction, we implemented a combined target prediction using three common prediction algorithms, miRanda, RNAhybrid and SeedMatch. miRanda [10] is an algorithm based on a dynamic programming alignment of microRNA and mRNA sequences, followed by a calculation of the duplex binding energy and filtering using empirically defined binding rules. RNAhybrid [34] is an algorithm for short nucleic acid hybridizations (free energy calculation) that uses estimated p-values to determine the significance of a predicted binding site and employs no empirical binding rules when filtering. SeedMatch is our own algorithm based on exact string matching of the microRNA seed region with the mRNA sequence similar to TargetScanS [2] or PicTar [7] that additionally searches for a perfect 3' seed to better reflect the binding patterns introduced by Brennecke et al. [9].

**Figure 3:**
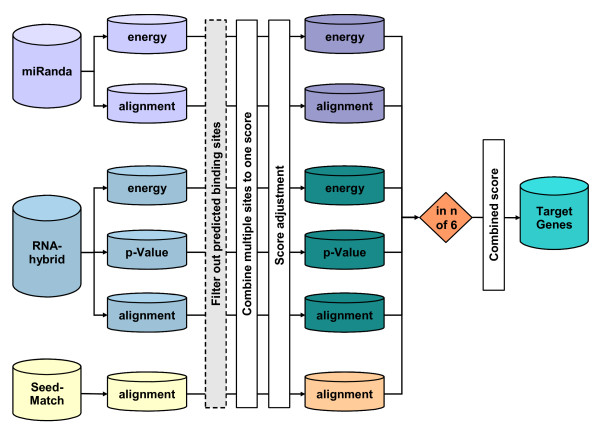
Work flow of target gene prediction. Three programs (miRanda, RNAhybrid, SeedMatch) were used separately to predict target sites. The energy score and p-values of RNAhybrid were supplemented by an alignment score calculated from the additionally provided alignment string. The overall six scores were treated as different predictors, motivated by a poor Pearson correlation (R^2 ^below 0.5) of the scores within each program. Within each input, the predicted target sites were filtered ("Classic", "Vari", "3000" filter) and combined to a final prediction of microRNA target sequences. For the resulting sets, the overlap of the different inputs was evaluated with an unweighted voting process. The parameter *n *of the voting process was set to four with an additional conservation filter, otherwise, the filter was set to five. A detailed description of target prediction is provided in Additional file 5.

Since the different target prediction programs are known to differ widely in the number of predictions and obtained gene lists [35-37], combining different predictors to produce an intersection or overlap of the different results would lead to an unacceptable loss of either sensitivity or selectivity [36]. Instead, we combined the prediction results using unweighted majority voting based on the predictions of complete transcripts (illustrated in Figure 3). We used three different strategies for selecting a prediction cutoff. First, the "Classic" filter assumes that a separation between functional and non-functional binding sites for each score is independent of a specific microRNA sequence and therefore uses the same cutoff values for all microRNAs. The "Vari" filter assumes a separation relative to a maximum score for the specific microRNA sequence. Lastly, the "3000" filter assumes that each mature microRNA sequence is available in a fixed copy number and restricts prediction results to the best 3000 target sequences. To validate our target gene prediction approach, we tested the recovery rate of predicted target genes in a set of 129 murine *mir-134 *targets, which were independently predicted and verified with luciferase-reporter assays by Miranda et al. [31]. A maximal, 10.665 fold enrichment was observed for non-conserved targets using the "Vari" filter (see Additional file 5 for a detailed description of the target gene prediction procedure and its validation).

**Figure 4:**
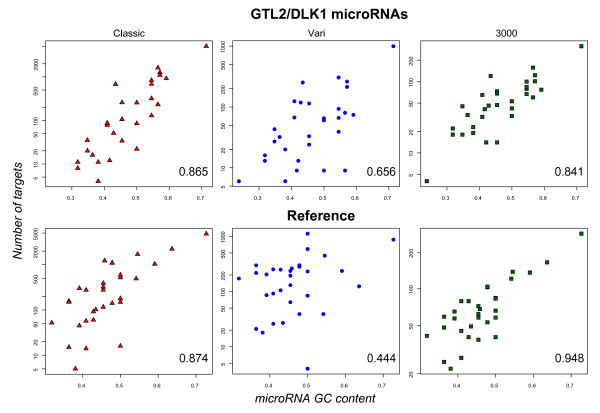
Relation between the mature microRNA's GC content and the number of predicted targets. The correlation of the GC content and the number of predicted targets was exemplified for predicted human targets of the *Dlk1/Gtl2 *microRNAs and the reference set that passed the conservation filter. The horizontal axis with the number of target genes is on logarithmic scale. Shown are the results for all three different target site filter strategies, Classic, Vari and 3000, with their corresponding Spearman's rank correlation coefficients. The Classic filter uses the same cutoff values for all microRNAs, the Vari filter uses cutoff values relative to a maximum score for each specific microRNA sequence and the 3000 filter only keeps the best 3000 predicted target sequences (see Additional file 5).

For all three filters, we observed that the GC content of microRNAs is highly correlated with the number of predicted targets (plots shown in Figure 4). This correlation likely arises from using sequence alignment and energy scores that favor GC matches over AT matches (higher number of hydrogen-bonds), and is consistent with the result that the lowest correlation is observed for the sequence-dependent "Vari" filter. In the following, we present the results from using the "Vari" filter, which reduces the GC effect by using cutoffs relative to the theoretically maximum score of each specific microRNA sequence.

For all microRNAs in the *Dlk1/Gtl2 *region, we observed on average 103 predicted targets conserved between human and mouse from an average 454 human targets. For the analyses reported below, we also looked for antitargets [13], which we define as all transcripts not contained in any of the raw predictions (lower cutoffs than used for the targets) for any of the used algorithms. The antitargets are, therefore, independent of the different filters used. We counted on average 332 antitargets conserved between human and mouse from 2925 human antitargets for the *Dlk1/Gtl2 *microRNAs.

### Targeting of repetitive elements

RNA interference should act as a defense mechanism against the transcription of retrotransposed sequences [38]. Since five microRNAs in the imprinted region are encoded by the antisense strand from the *Retrotransposon-like 1 *gene, we asked whether the microRNAs derived from this imprinted region generally target retroelements in the human or mouse genome. We predicted microRNA binding sites for 868 repetitive human sequences and 585 mouse sequences [39]. Predominantly, Endogenous Retrovirus sequences, some Non-Long Terminal Repeat (LTR) Retrotransposon, LTR Retrotransposon, and DNA transposon elements were predicted targets of the *Dlk1/Gtl2 *microRNAs. We did not predict Variable Number Tandem Repeats like (TAGA)_n_, (GACA)_n_, or (CGAA)_n _as targets of this microRNA cluster. The number of predicted Satellite sequences, not further classified Interspersed Repeats and other repeats was very low. By analyzing the enrichment for these repeat types, we found that the *Dlk1/Gtl2 *microRNAs particularly target Endogenous Retrovirus and L1 elements. This effect is not restricted to the *anti-Rtl *microRNAs with perfect complementarity to the retrotransposon *Rtl1 *(Figure 1) [17,40].

### Overlap of predicted target genes

The target gene predictions enabled us to analyze the degree of similarity among the target gene sets for microRNAs within the *Dlk1/Gtl2 *locus. This evaluation reveals whether high sequence similarities among the microRNA hairpins in this region actually result in gene co-regulation. For this purpose, we defined a measure of overlap in two ways. First, we measured overlap using the number of common genes divided by the number of unique genes in the two sets. Since this definition penalizes large differences between the number of targets, we also defined overlap as the fraction of common targets in the smaller set of predicted microRNA targets. This second definition completely ignores differences between the number of targets; therefore, microRNAs with small target sets are easily scored as very similar to microRNAs with large target sets. When mouse and human mature microRNAs were plotted graphically (data not shown), we observed large hubs representing microRNAs with large target sets of mainly GC-rich mature microRNAs, which agglomerated satellites of microRNAs with small target sets.

Using the "Vari" filter strategy and predicted human targets, the highest overlap for the first measure is only 26.2% (10.0% for conserved targets), whereas the second measure results in an overlap of up to 48.8% (55.6% for conserved targets). With respect to the microRNA sequence families defined above, we observe that mature sequences must be highly similar to show a considerable overlap in their targets. For example, the sequences with the highest overlap of 26.2%/48.8% (*hsa-miR-376a *and *hsa-miR-376b*) differ in only two bases (one in length and one base substitution). Thus, the overlap in the identified target genes results primarily from using the same sequence motifs as binding sites. For the reference set, the highest target overlap is 38.3% and 62.3% (38.6% and 57.4% conserved targets) for the first and second overlap definition respectively. The corresponding sequences, *hsa-miR-103 *and *hsa-miR-107*, show only one base substitution.

**Figure 5:**
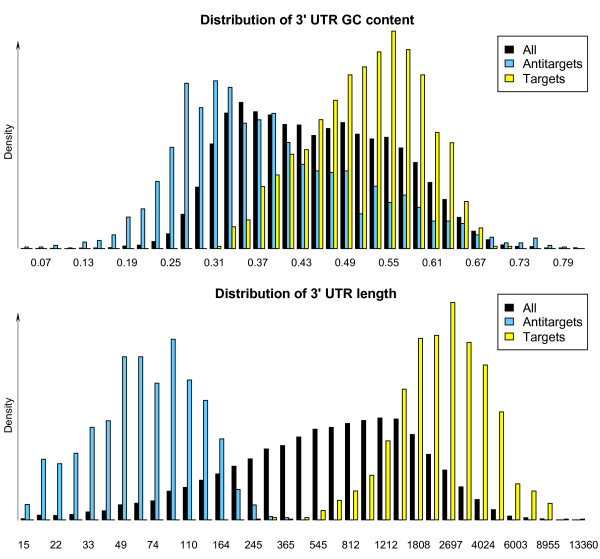
GC content and length distribution of 3' UTR sequences. Shown is the density distribution of GC content and length in the set of all 3' UTR sequences, in the set of predicted human "Vari" targets of at least three *Dlk1/Gtl2 *microRNAs and in the set of human antitargets of at least 16 *Dlk1/Gtl2 *microRNAs. The frequencies of the bins are normalized to equal one for each set of sequences (density distribution), which corrects for the different number of sequences in each set. For a better visualization of the 3' UTR length, the natural logarithm of the length was used.

In addition to exploring the target and antitarget lists of individual microRNAs, we also analyzed the merged target and antitarget lists for both the *Dlk1/Gtl2 *microRNAs and the randomly selected reference set. This analysis facilitates a further investigation for a common function of the microRNAs in the imprinted cluster. To reduce the impact of transcripts only predicted for a small subset of microRNAs, we removed all sequences that were predicted for fewer than three microRNAs from the merged target lists and fewer than 16 microRNAs from the merged antitarget lists. In analyzing these targets and antitargets, we notice that the average target 3' UTR length is about 2829 nt in human (2870 nt in mouse) with the "Vari" filter – three times greater than the average 3' UTR length of all transcripts analyzed – and that the GC content of the targets is about 6.5% higher in human (4.0% in mouse) than the average. For the antitargets, we see the same shift in the other direction for the 3' UTR length and GC content (Figure 5). These observations are not unexpected given the known preference of target predictors for guanine and cytosine bases and that long sequences have a higher probability of containing target sites. Similar results were obtained for the targets and antitargets of the reference set.

### Chromosomal distribution of predicted target genes

Imprinting regions, tandem duplications in the *Hox *gene families and clustering of house-keeping genes are good examples of genes that are not uniformly distributed over the genome. At the chromosomal level, genes expressed in somatic testis cells accumulate on the X chromosome [41]. To evaluate whether target genes of microRNAs cluster on distinct chromosomes due to co-regulation, co-function or a common evolutionary history [42], we analyzed the chromosomal distribution of the predicted target genes in our dataset. The results show a highly non-uniform target gene distribution for the microRNAs within both the *Dlk1/Gtl2 *region and the reference set. For the microRNAs in the *Dlk1/Gtl2 *region, some chromosomes have a notably high target density (e.g. human chromosomes 9, 15, 17, and 22), some a rather small density (e.g. 2, 4, 12, and X) and a majority around the average. For the reference set, we observed similar chromosomal distributions.

**Figure 6:**
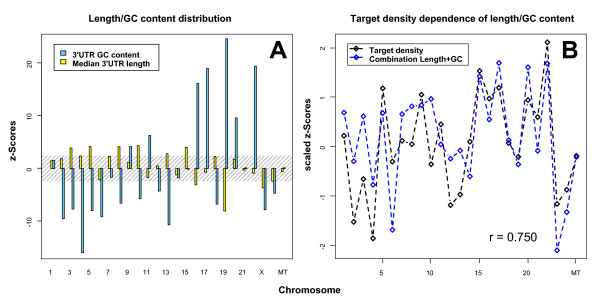
Chromosomal distribution of predicted target genes is influenced by length and GC content of 3' UTRs. The line chart of the z-scores of human chromosomal 3' UTR GC and length distribution (A) and the line chart of the combination of these z-scores compared to the human "Vari" target density z-scores of at least three *Dlk1/Gtl2 *microRNAs (B). The scattered area in (A) corresponds to a p-value higher than 0.05. The values in (B) are normalized by their standard deviation to focus on the relationship and not the values. Additionally, the Pearson correlation coefficient r is given. The plots show that the observed non-uniform distribution of the targets is probably caused by the non-uniform distribution of 3' UTR GC content and length over the chromosomes. For predicted murine targets a similar effect is observed, the corresponding Pearson correlation coefficient is 0.950. If we restrict the chromosomes included in the calculation to the chromosomes with target densities significantly different to a random distribution (p-value smaller than 0.01), we obtain even higher values (0.982 and 0.968 respectively).

Mammalian genomic regions can differ considerably in their GC contents, which may bias the chromosomal distribution of potential microRNA target genes. Therefore, we compared both the total 3' UTR GC content (concatenation of the sequences) and the median 3' UTR length of the targets within each chromosome to the distribution of the same number of randomly selected sequences from the same chromosome. All three filter strategies, show target selection (in many cases significant with a p-value < 0.01; sampling test, data not shown) based on their 3' UTR sequence length and GC content within each chromosome. Testing whether the sequence annotation to chromosomes is consistent with a random model of gene distribution, seven human chromosomes show significantly increased 3' UTR GC content, and twelve significantly decreased GC content. Similarly seven human chromosomes exhibit a significantly increased median 3' UTR sequence length and four show a significantly decreased length (Figure 6A). Comparing the target density, the median 3' UTR length, and the GC content for each chromosome, we observed that the z-scores of the target density follow the linear combination of the z-scores of the median 3' UTR length and the GC content (Figure 6B). Calculating Pearson correlation coefficients to substantiate the visual impression, we obtain 0.945 for mouse and 0.750 for human. These calculations show that the observed non-uniform target distribution is likely attributable to the non-uniform distribution of 3' UTR GC content and length over the chromosomes. Using antitargets for the corresponding analyses, we obtain inverse results (in agreement with the inverse definition of antitargets).

Imprinted genes may interact with each other within a regulatory network [43]. Co-regulation of these genes could be mediated by common regulatory elements in imprinted regions or by imprinted microRNAs. Thus, imprinted regions might show enrichment of predicted target genes. Based on this assumption we specifically analyzed genes in imprinted regions. We found no evidence for significant enrichment or depletion of imprinted genes in the groups of predicted target or antitarget genes. Also, the median 3' UTR lengths (human 837 nt, mouse 1154.5 nt) and GC contents (human 39.76%, mouse 41.90%) were similar to other genes in the human and mouse genome (Wilcoxon rank sum test with continuity correction, all p-values > 0.05).

Genomic imprinting is a mechanism of gene regulation that only occurs in therian species. There is evidence that imprinted gene expression in the *Dlk1*/*Gtl2 *region was established after divergence of the marsupial and eutherian lineages, at the time when the microRNAs encoding sequences in the region were amplified [28,44,45]. Since species divergence is related to changes in protein sequences and changes in gene expression, a special evolutionary relationship between eutherian imprinted microRNAs and eutherian-specific genes appears likely.

Addressing this question, we tested if eutherian-specific genes are enriched among the predicted microRNA target genes. For this purpose, we identified 198 human genes without orthologs in any fish, bird, marsupial, or monotreme genomes, but shared among eutherians ([46], for details see Material and Methods). Eutherian-specific genes are not enriched among the predicted target genes, but they show enrichment within the groups of antitargets, i.e. genes that show a depletion of microRNA-binding sites (2.48 fold enrichment in non-conserved antitargets, hypergeometric test p-value < 0.0001). This effect is also observed in the antitargets of the reference set (2.7 fold enrichment in non-conserved antitargets, hypergeometric test p-value < 0.0001). This enrichment in the antitarget sets might be due to a significantly reduced 3' UTR length (median 245.5 nt, Wilcoxon rank sum test with continuity correction, p-value < 2.2e-16) of the eutherian-specific genes compared to all human transcripts used for analysis.

### Expression of predicted targets

**Figure 7:**
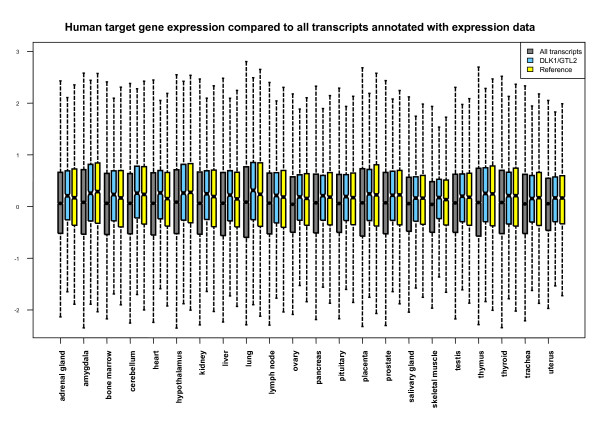
Gene expression of predicted target genes compared to all transcripts with annotated expression data. The figure shows a box plot of the expression values of predicted human "Vari" targets of at least three microRNAs within the *Dlk1/Gtl2 *region and randomly selected microRNAs of the reference set compared to the background (the expression values of all transcripts with annotated expression data).

Tissue-specific microRNA expression patterns and non-translated RNAs encoded by the imprinted *Dlk1/Gtl2 *region have been analyzed in most detail in mouse, in which these RNAs are predominantly expressed in brain, neonatal limbs and placenta [19,20]. Imprinting mutations in the region cause a number of different abnormalities that affect the skeletal muscles, skeleton and placenta [23,24]. Thus, the expression patterns of potential target genes might highlight possible roles for aberrant target gene expression in the observed abnormalities. To analyze the expression of the predicted targets, we combined clustered heat maps, correlation maps and box plots to a condensed version of the GNF gene expression atlas of mouse and human protein-encoding transcripts [47]. In addition to these exploratory techniques, we compared the median gene expression value and the variance of the expression values of the predicted targets in each tissue to the distribution obtained for equally-sized random transcript selections. This analysis revealed higher median values and significantly lower variances in the expression values of the predicted targets in each tissue (see Additional file 6). These two effects are also visible in the box plots (Figure 7) and are independent of the set of microRNAs used (*Dlk1/Gtl2 *or reference set).

**Figure 8:**
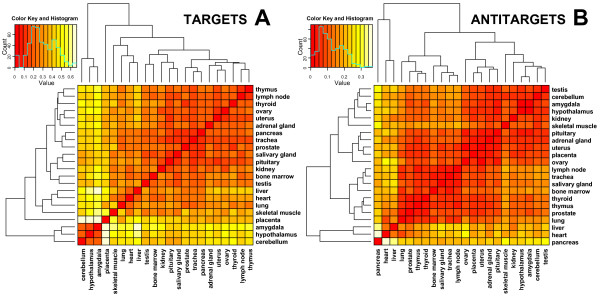
Correlation map of predicted human "Vari" target transcripts (A) and antitarget transcripts (B) with annotated expression data. The figure shows the correlation map (squared Pearson correlation) of the expression values in each tissue. The expression values of the targets (A) show a clear separation of the three brain tissues amygdala, cerebellum and hypothalamus, from the heterogeneous group of other tissues. This separation is not observed for the antitargets.

When analyzing tissue-specific gene expression patterns, we observed a separation of the three brain tissues amygdala, cerebellum and hypothalamus from the heterogeneous group of other tissues (Figure 8A). This result was not specific for the targets of the imprinted *Dlk1/Gtl2 *microRNAs, which might be expected given the strong expression of these microRNAs in brain. It also applies to the target genes of the reference set. In fact, this separation of the three brain tissues appears to be a common feature of the target gene expression over several tissues [48]. The relevance of these observations is supported by the fact that the variance in antitarget expression is significantly higher (rather than lower) and that no separation of brain tissues was observed for the antitargets (Figure 8B). The shift in median gene expression values and the variance of expression values is also observed for sequences only selected by 3' UTR sequence length and GC content. For a high GC content and high sequence length, a higher median and lower variance is observed, and for low GC content and length, a lower median – observations similar to the targets and antitargets respectively.

### GO annotation of predicted targets

Imprinting mutations within the *Dlk1*/*Gtl2 *region are associated with a broad spectrum of phenotypic abnormalities affecting the regulation of growth and the morphology of placenta, the maturation of muscle fibers and the ossification processes in bones [23,24]. The potential involvement of predicted microRNA target genes in specific biological processes might highlight the impact of deregulated microRNAs on the observed phenotypes. Mining for over- and underrepresented Gene Ontology (GO) terms [49] provides a well-established and scalable way to identify common functional roles among sets of genes. Briefly, the GO is based on the annotation of the biological process, the molecular function and cellular localization of the translated proteins that correspond to the gene of interest. Over- and underrepresented GO biological process terms were determined for the predicted targets and antitargets (using conditional hypergeometric tests as implemented in the GOstats bioconductor package [50]). The over-represented terms for the target genes of the microRNAs within the *Dlk1/Gtl2 *region (Table 1) include developmental process, anatomical structure development, cell communication, regulation of biological process, transport, phosphorus metabolic process and cell adhesion. Under-represented processes include cellular metabolism, electron transport, protein biosynthesis and metabolism, sexual reproduction, response to a biotic stimulus (like bacteria and pathogens), DNA and RNA metabolism and RNA processing. Again, we observed similar results for the reference set and inverse results for the antitargets.

**Table 1:** GO annotation of predicted *Dlk1/Gtl2 *microRNA targets.

**GOBPID**	**p-value**	**Count**	**Enrich.**	**Term**
***Over-represented terms***				

GO:0035023	3.442E-04	14/66	2.802	regulation of Rho protein signal transduction
GO:0007156	3.159E-05	24/129	2.458	homophilic cell adhesion
GO:0007264	2.171E-04	42/315	1.761	small GTPase mediated signal transduction
GO:0009966	3.742E-04	41/313	1.730	regulation of signal transduction
GO:0007155	1.052E-05	74/593	1.648	cell adhesion
GO:0007154	8.479E-05	256/2768	1.222	cell communication

***Over-represented terms (conservation filter)***				

GO:0015804	7.198E-04	3/11	16.037	neutral amino acid transport
GO:0046942	1.492E-04	6/48	7.350	carboxylic acid transport
GO:0015837	8.475E-04	5/44	6.682	amine transport

***Under-represented terms***				

GO:0006281	5.368E-04	4/199	0.266	DNA repair
GO:0006396	4.214E-04	13/383	0.448	RNA processing
GO:0050896	4.164E-04	105/1836	0.755	response to stimulus
GO:0044237	5.674E-04	416/6089	0.902	cellular metabolism
GO:0007582	6.463E-05	682/9485	0.950	physiological process

***No under-represented terms (conservation filter)***				

Over- and under-represented GO "biological process" terms of human "Vari" targets of at least three microRNAs. The significance cutoff was set to a p-value of 0.001.

To investigate whether 3' UTR length and GC content might also correlate with specific biological processes, we repeated our GO analysis using a selection of the longest and GC-richest 3' UTRs, as well as on the shortest and GC-poorest 3' UTRs. We observed highly similar GO terms, indicating that these biological processes are correlated with the sequence signature of the 3' UTRs.

## Discussion

The results of our microRNA sequence analysis show that microRNAs encoded within the *Dlk1/Gtl2 *region are well conserved between mouse and human, both in terms of their pre-microRNA and mature sequences. A large fraction of the microRNAs in this imprinted cluster is contained in large families of hairpin sequences. This is true for the microRNAs clustering between *Rian *and the 3' end of *Mirg*, thereby confirming previous studies that propose multiple tandem duplications of an ancestral precursor sequence as the evolutionary origin of these pre-microRNAs [19,26-28]. However, we observed that not a single pre-microRNA between *Gtl2 *and *Rian *is member of a hairpin family and that there is also no mature sequence family between *Gtl2 *and *Rian *or spanning over the whole region from *Gtl2 *to the 3' end of *Mirg*. Hence, we see little indication that microRNAs in the upstream region of *Rian *originate from the same ancestral microRNA as the microRNAs downstream of *Rian*.

Considering the pre-microRNA sequence family composition, a high similarity among the mature microRNAs would be also expected. Surprisingly, unlike the pre-microRNAs, mature sequences in the downstream region of *Rian *are rather divergent, a feature that has not yet been addressed. We observe fewer similar mature sequences and therefore, smaller mature microRNA families. Since the mature sequences are well conserved between mouse and human and the orthologous pre-microRNAs also encode the most similar mature sequences, the divergence of these microRNAs is likely to date more than 75 million years ago, to a common ancestor shared by mouse and human [51]. From our observations, we suggest the following hypothesis: After the sequence duplication event in a common ancestor, selective pressure on the duplicates was sufficiently low to enable the pronounced diversity to evolve rapidly. This divergence later became functional and preserved in descendants.

Our analysis of sequence similarities between all mature and pre-microRNA sequences within each species revealed that the *Dlk1/Gtl2 *microRNAs show only weak sequence similarities to other microRNAs in the genome. This supports our hypothesis that this cluster is structurally distinct from other microRNAs in both the human and mouse genome. Possibly as a consequence of mature microRNA sequence divergence, the predicted microRNA target genes are diverse. Our analysis showed very little overlap between individual microRNAs in the target lists. Even smaller sequence families defined on the mature microRNAs did not exhibit considerable overlaps in predicted targets, which could have hinted at a concerted specialized function of these microRNAs.

We observed a high correlation between the GC content of the mature microRNA and the number of predicted targets, which we attributed to the greater contribution of GC base pairing over AT base pairing in determining sequence and energy scores during the target prediction process. While the biological relevance of this effect cannot be fully assessed in the absence of appropriate experimental data, we could derive a target prediction method with cutoffs that depend on the mature microRNA sequence using the "Vari" filter method. This reduces the GC correlation and appears to offer a good compromise between sensitivity and specificity (see Additional file 5). Similarly, the GC content and length of the 3' UTR target sequences was observed to influence target gene prediction. We demonstrated that the chromosomal densities of target sequence predictions correlate with GC content and the length of 3' UTRs of the genes on each chromosome. Consequently, some chromosomes are enriched or depleted for microRNA target genes.

As might be expected for mature microRNAs that do not show pronounced similarities, the microRNAs derived from the imprinted region target a broad spectrum of genes. This observation does not support that these clustered microRNAs fulfill one specific function. However, their tissue-specific expression pattern suggests that they might be able to simultaneously reduce the expression of numerous genes in distinct cell-types.

Since the microRNAs analyzed in this study are derived from an imprinted region their possible interactions in regulatory networks with one another are of particular interest. In our study, we find no evidence for a significant enrichment or depletion of imprinted genes among the predicted target or antitarget genes. Thus, a specific down-regulation of imprinted genes is probably not one of the major functions of the microRNAs within the *Dlk1/Gtl2 *region.

Unlike the imprinted genes, we find an enrichment of eutherian-specific genes within the predicted antitarget sets. This effect is apparently associated with the short 3' UTRs of eutherian-specific genes. The short length of the 3' UTRs might be a feature caused by specific evolutionary origins of these genes, such as possible retro-transposition or tandem-duplication events. Due to their late occurrence in evolution, these genes might also have been unable to accumulate an equal amount of microRNA-binding sites. Alternatively, there might be evolutionary selection against microRNA binding sites in recently evolved genes, linked to an ongoing process of acquiring specific functions. The enrichment of eutherian-specific genes is also observed for the antitargets of the randomly selected microRNAs in the reference set. Thus, the observed effect is not due to special associations between the location of the microRNAs within the *Dlk1/Gtl2 *imprinting region and eutherian-specific genes in terms of their function or shared evolutionary history.

The analysis of gene expression data and functional annotations revealed general characteristics of microRNA target genes. Predicted microRNA targets exhibited enhanced expression with reduced variation in expression values among the targeted transcripts in each tissue. Our observation is consistent with the model that post-transcriptional regulation by microRNAs leads to a stabilization of expression levels, rather than a complete silencing of target genes [52]. The target genes also exhibit characteristic GO annotation terms, including development, regulation and cell communication processes, which are in total agreement with previous studies of microRNA target genes [2,13,53,54]. Following the observation of the selection for long and GC-rich transcripts in microRNA target gene prediction, we noticed that sets of transcripts only selected by the length and GC content of their 3' UTR also yield similar results in gene expression and GO annotation. The agreement between our results and previous findings suggests a relation between the length and GC content of a 3' UTR sequence and the biological function of the gene. Therefore, we conclude that the differences in the sequence properties of 3' UTR sequences might be due to specific biological functions that depend on the effects of post-transcriptional regulation like a microRNA mediated stabilization of gene expression levels.

## Conclusion

Although the results of our microRNA sequence analysis showed that the imprinted *Dlk1/Gtl2 *microRNAs are well conserved between mouse and human, we find that the mature microRNAs are quite distinct among each other in terms of their sequences and of the genes they are predicted to regulate. Co-expression of such a high number of microRNA genes may provide an efficient way for boosting different mature microRNAs in a particular cell type, thereby influencing the activity of a high number of genes simultaneously. Although there is no obvious relation to the observed phenotypes for imprinting mutations within the imprinted *Dlk1/Gtl2 *region [23,24], susceptible developmental processes can be substantially disturbed due to an altered tissue-specific co-expression of those microRNAs. Considering the observed diversity of the microRNAs and their broad spectrum of potential targets in protein-coding genes and repetitive elements, these microRNAs might represent an important modulator of tissue-specific gene expression and therefore, an integral part of the microRNA gene regulation network in mouse and human.

## Methods

### 3' UTR sequences and sequences of repetitive elements

The 3' UTR sequences for mouse and human were downloaded using the UCSC Table Browser data retrieval tool [55] from the UCSC Genome Bioinformatics Site. Tables were created from Known Genes/knownGene with the fields name, chrom, strand, txStart, txEnd, proteinID, foldUTR3.name and foldUTR3.seq. The Feb. 2006 assembly (NCBIM36/mm8) was used for mouse and the Mar. 2006 assembly (NCBI 36.1/hg18) for human. Target gene predictions were performed on 38,316 human and 30,905 murine non-redundant identifiers. For chromosomal distribution analysis, the set was restricted to identifiers properly assembled to a chromosomal region giving rise to 38,153 human and 30,796 mouse IDs. Using the tables Known Genes/hgBlastTab and Known Genes/mmBlastTab provided by the UCSC Table Browser, we identified 14,662 orthologous transcripts that are unambiguously mapped between human and mouse.

We neither used a fixed number of bases where no 3' UTR annotation was available nor extended short 3' UTR sequences as is sometimes done to reduce heterogeneity in the annotated 3' UTR sets. 66.98% of the human and 67.04% of the mouse 3' UTR sequences used for evaluation show one of the most common polyadenylation signals (AAUAAA and AUUAAA) in the last 100 sequence bases. The average 3' UTR length of these sets is 1,003 bases in human and 956 bases in mouse. Furthermore, we did not mask repeats in the sequences, because microRNAs can target repetitive elements [56]. Additionally, repetitive DNA elements, including retroelements, were retrieved from Repbase Update [39] for target gene prediction. We obtained FASTA files for mouse (containing 585 sequences) and human (containing 868 sequences).

### MicroRNA sequences

Pre-microRNA sequences and mature microRNA sequences used for the genome-wide study of sequence similarities were obtained from miRBase [21] version 9.0. Mature microRNA sequences used for prediction (see Additional file 1) were obtained from miRBase version 8.0. There are 31 mature *Dlk1/Gtl2 *sequences available in human and mouse, as well as 14 sequences only available in human and 12 only available in mouse. The microRNAs of the reference set were selected as follows: from 199 mature microRNA pairs with a SQ ≥ 0.9 and a pre-microRNA identifier shared between mouse and human, 31 conserved microRNAs were randomly selected. 14 human and 12 mouse non-conserved microRNAs were randomly selected from miRBase identifiers available in only one species.

### Pairwise sequence and multiple sequence alignments

All alignments were created with a downloaded version of the alignment tool ClustalW 1.83 [33], obtained from . The program was used with its default parameters. For sequence similarity comparisons the similarity quotient SQ was established as the ClustalW alignment score of two sequences divided by the minimum of the alignment scores for each sequence with itself. This quotient has its maximum of 1, which occurs if both sequences are equal over the length of the shorter sequence and its minimum of 0, if the alignment score for the sequence pair is 0.

### Target gene prediction

A detailed description of the combination approach, the SeedMatch algorithm and the parameters used is available as supplementary text (see Additional file 5).

### Expression data for mouse and human

A preprocessed version of the gene expression atlas of mouse and human protein-encoding transcripts [47] was kindly provided by C. Steinhoff (Max-Planck-Institute for Molecular Genetics, Berlin). The arrays used (GNF1H and GNF1M) are custom-designed whole genome gene expression arrays. A map of their identifiers onto the human and mouse RefSeq/EMBL sequence identifiers is available using the UCSC Table Browser data retrieval tool [55]. For human (Mar. 2006) the table Affy GNF1H/knownToGnfAtlas2 and for mouse (Feb. 2006) the table Affy GNF1M/knownToGnf1m was retrieved.

The data [47] was preprocessed by the Max-Planck-Institute of Molecular Genetics as follows (C. Steinhoff, pers. comm.): For normalization of raw probe set intensities, the calibration and variance stabilization method [57] (VSN) was used. It was assumed that in each experiment most genes were not differentially expressed in comparison to all other experiments on the same array type. For the VSN model, parameters were estimated on a random subset of 50% of the probes and used to transform the entire array. Applying the median polish method [58] after normalization, the probe-set intensities of each probe set were summarized. Hereby, a robust additive model was fitted across the arrays for each probe-set. Further, a full linear model was fitted to the data [59] by specifying the corresponding parameters. Repeated experiments were averaged and only experiments annotated with tissues for human and mouse were considered for further analyses.

The gene expression data of the predicted 3' UTR target sequences and antitargets was evaluated using R 2.4.1 [60] and the gplots 2.3.2 package. To investigate an effect of the microRNAs on gene expression, the median expression value of the predicted targets and antitargets, as well as the variance within each tissue, were calculated. 1,000 equally-sized sets of randomly chosen transcripts were used to obtain the distribution of these features. The distributions exhibit close to normal distribution with a mean μ and standard deviation σ. Therefore, z-scores (z = (x-μ)/σ) and corresponding p-values were calculated for the values obtained from the targets and antitargets. To examine a possible interrelation with the target prediction preference for long and GC-rich sequences, the 1,000 longest and GC-richest, as well as the 1,000 shortest and GC-poorest transcripts, were tested. For this purpose, normalized and centered 3' UTR GC content and length values of 3' UTR sequences with available expression data were totaled, and the new value was used for sequence selection.

### Chromosomal gene distribution

The 3' UTR sequence identifiers obtained were annotated with a chromosomal position. For non-unique chromosomal coordinates (chromosome name, strand, start and end position of the transcript), only the longest 3' UTR sequence was kept, to rule out a possible bias caused by multiple annotated sequences for the same locus. The number of targets over the number of sequences (target density), as well as the number of antitargets over the number of sequences (antitarget density), were evaluated for each chromosome and data set. These densities were compared to sets of randomly distributed targets or antitargets. For the random distributions, an equal number of targets and antitargets were randomly chosen a thousand times from the set of sequence identifiers. Close to normal distributions were obtained for each chromosome. With these values, chromosome-specific z-scores and corresponding p-values were estimated for the determined target and antitarget densities using R 2.4.1 [60].

### GC content and length distribution

The assumption that microRNA target prediction programs select for long and GC-rich 3' UTR sequences was empirically verified using the length and GC content distribution of targets and antitargets. To evaluate chromosomal effects, the median length and overall GC content of the predicted targets and antitargets on each chromosome were compared to 1,000 randomly selected sets with the same number of 3' UTR sequences from the same chromosome. These random sets exhibited close to normal distribution and were used to determine the mean and standard deviation for the z-score and p-value calculations as described above. Additionally, target and antitarget densities obtained for each chromosome were compared to the GC content and sequence length of the 3' UTR sequences on each chromosome. This was done by calculating the overall GC content of the concatenation of all 3' UTR sequences on each chromosome and relating it to the distribution in 1,000 randomly selected sets with the same total number of sequences per chromosome chosen from all chromosomes. The same analysis was performed for the median length of the 3' UTR sequences on each chromosome, and z-scores and p-values were estimated for both sequence properties. The results were variance normalized and then added to retrieve the observed target and antitarget densities in Figure 6. R 2.4.1 scripts were used for these analyses of length and GC content.

### Analysis of Gene Ontology annotation data

For the analysis of Gene Ontology (GO) terms [49], the sequence identifiers were mapped onto Entrez Gene IDs using tables of the UCSC Table Browser data retrieval tool [55] and custom Python scripts. A map of human (Mar. 2006) and mouse (Feb. 2006) RefSeq/EMBL sequence identifiers was obtained from the tables Known Genes/knownToLocusLink for each organism. The analysis of over- and under-represented Gene Ontology terms was performed using the GOstats package from the Bioconductor project BioC 2.0 [50] (containing Entrez annotation data in the humanLLMappings and mouseLLMappings packages) with R 2.5.0 [60]. We used the conditional hypergeometric test of the GOstats package, which incorporates the relationship among the GO terms into the significance test, similar to the approach presented by Alexa et al. [61]. For the predicted targets and antitargets in the 3' UTR sequence set, over-represented and under-represented terms were calculated with all annotated Entrez IDs as background. The test was performed for the GO category "biological process" with a significance cutoff of 0.001. To examine a possible interrelation between the annotated biological function and the preference of target predictions for long and GC-rich sequences, the 1,000 longest and GC-richest, as well as the 1,000 shortest and GC-poorest transcripts were tested for over-represented and under-represented terms with the same significance cutoff. To select equally for GC content and length, the normalized and centered 3' UTR GC content and length values were totaled, and the new value was used for sequence selection.

### Imprinted genes

Imprinted transcripts were selected for analysis from the Catalogue of Imprinted Genes [62]. Transcripts with marginal experimental support were excluded. The final set of 31 human transcripts and 36 murine transcripts is listed in Additional file 7. Hypergeometric tests and Wilcoxon rank sum tests with continuity correction were performed using using R 2.4.1 [60].

### Eutherian transcripts

InParanoid Eukaryotic Ortholog Groups (version 6.0, August 2007) [46] were used to identify human proteins shared only among eutherians. These proteins were identified by calculating the intersection of human proteins shared with *Bos taurus*, *Canis familiaris*, *Macaca mulatta*, *Mus musculus*, *Pan troglodytes *and *Rattus norvegicus*. From this intersection all proteins shared with at least one species out of *Aedes aegypti*, *Anopheles gambiae*, *Apis mellifera*, *Caenorhabditis briggsae*, *Caenorhabditis elegans*, *Caenorhabditis remanei*, *Ciona intestinalis*, *Danio rerio*, *Drosophila melanogaster*, *Drosophila pseudoobscura*, *Gallus gallus*, *Gasterosteus aculeatus*, *Monodelphis domestica*, *Takifugu rubripes*, *Tetraodon nigroviridis*, or *Xenopus tropicalis *were removed. The remaining proteins were mapped to transcript identifiers, and a final list of 198 proteins was used for analysis (see Additional file 8). Hypergeometric tests and Wilcoxon rank sum tests with continuity correction were performed using R 2.4.1 [60].

## Authors' contributions

The predictions and analyses were performed by MK with substantial input by MP and CB. The manuscript was written by MK, CB and MP. All authors read and approved the final manuscript.

## Supplementary Material

Additional file 1 Mature microRNAs used for target gene prediction. Click here for file

Additional file 2 Murine similarity graphs. Click here for file

Additional file 3 Alignment of miRBase *hsa-miR-154 *family. Click here for file

Additional file 4 Pair-wise mature sequence alignments. Click here for file

Additional file 5 Detailed description of target prediction approach and validation. Click here for file

Additional file 6 Gene expression analysis of human targets. Click here for file

Additional file 7 List of imprinted genes in mouse and human used for analysis. Click here for file

Additional file 8 List of eutherian-specific human proteins used for analysis. Click here for file
