# Supplementary material for: Structural conservation versus functional divergence of maternally expressed microRNAs in the Dlk1/Gtl2 imprinting region
Source: BMC Genomics. 2008 Jul 23;9:346. doi: 10.1186/1471-2164-9-346 (PMC2500034; doi:10.1186/1471-2164-9-346)
Supplement: Additional file 1 — Mature microRNAs used for target gene prediction. [file 1471-2164-9-346-S1.pdf]

Supplementary table S1: miRBase mature sequence identifiers from the *Dlk1/Gtl2* region and the reference set used for target gene prediction. In both sets, there are 31 mature microRNA identifiers available for human and mouse as well as 14 available only for human and 12 available only for mouse. The microRNAs in the reference set were randomly selected as follows: 31 conserved microRNAs were selected from 199 mature microRNA pairs with an SQ $\geq$ 0.9 and a pre-microRNA identifier shared between mouse and human. 14 human and 12 mouse non-conserved microRNAs were selected from miRBase identifiers available for only one species.

**DLK1/GTL2 region**	
Human (con.) [hsa-miR-]	127, 134, 136, 154, 299-5p, 323, 329, 337, 369-3p, 369-5p, 370, 376a, 376a*, 376b, 377, 379, 380-3p, 380-5p, 381, 382, 409-3p, 410, 412, 433-3p, 431, 485-3p, 485-5p, 487b, 494, 495, 539
Mouse (con.) [mmu-miR-]	127, 134, 136, 154, 299, 323, 329, 337, 369-3p, 369-5p, 370, 376a, 376a*, 376b, 377, 379, 380-3p, 380-5p, 381, 382, 409, 410, 412, 433-3p, 431, 485-3p, 485-5p, 487b, 494, 495, 539
Human (non-con.) [hsa-miR-]	154*, 299-3p, 368, 409-5p, 411, 432, 432*, 453, 487a, 496, 544, 654, 655, 656
Mouse (non-con.) [mmu-miR-]	300, 341, 376b*, 376c, 411, 433-5p, 434-3p, 434-5p, 540, 541, 543, 679
**Reference set**	
Human (con.) [hsa-miR-]	10a, 15b, 17-3p, 17-5p, 22, 25, 26a, 103, 107, 132, 135b, 141, 143, 155, 181a, 181a*, 185, 186, 194, 195, 205, 215, 301, 324-3p, 342, 361, 424, 429, 486, 490, let-7f
Mouse (con.) [mmu-miR-]	10a, 15b, 17-3p, 17-5p, 22, 25, 26a, 103, 107, 132, 135b, 141, 143, 155, 181a, 181a*, 185, 186, 194, 195, 205, 215, 301, 324-3p, 342, 361, 424, 429, 486, 490, let-7f
Human (non-con.) [hsa-miR-]	181d, 220, 513, 518a, 525, 545, 551a, 555, 556, 578, 580, 589, 601, 662
Mouse (non-con.) [mmu-miR-]	101a, 201, 351, 546, 670, 675-5p, 677, 680, 688, 689, 700, 717
