# Supplementary material for: Structural conservation versus functional divergence of maternally expressed microRNAs in the Dlk1/Gtl2 imprinting region
Source: BMC Genomics. 2008 Jul 23;9:346. doi: 10.1186/1471-2164-9-346 (PMC2500034; doi:10.1186/1471-2164-9-346)
Supplement: Additional file 2 — Murine similarity graphs. [file 1471-2164-9-346-S2.pdf]

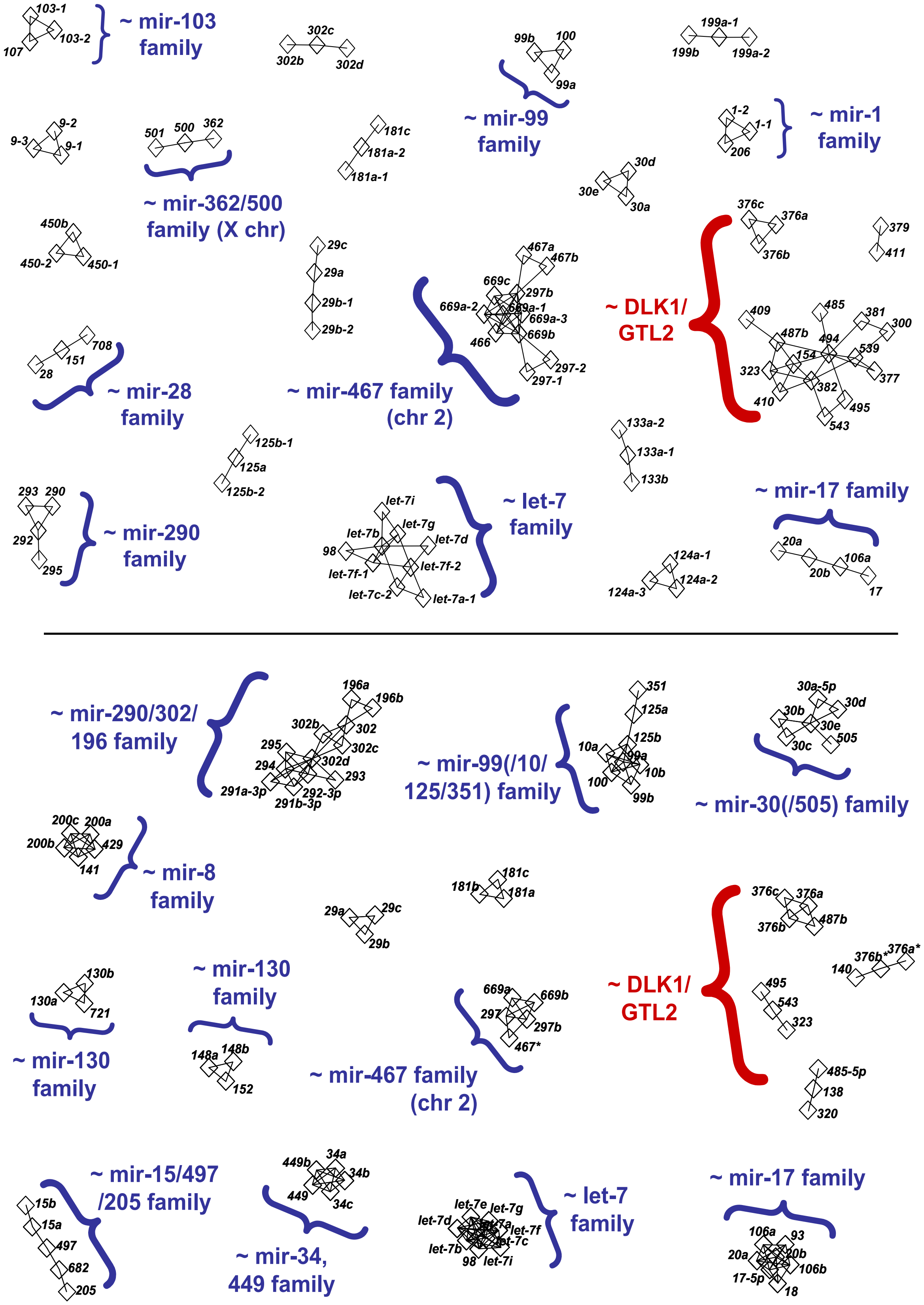

Figure S1: Murine microRNAs in the *Dlk1/Gtl2* region exhibit unique sequence characteristics. *Top:* Graph of mouse microRNA sequence similarities based on complete hairpin sequences, restricted to components with more than two nodes. *Bottom:* Graph of mature microRNA sequence similarities for mouse, restricted to components with more than two nodes. Components of heterogeneous mature microRNA identifiers are annotated based on miRBase data.
