# Supplementary material for: Structural conservation versus functional divergence of maternally expressed microRNAs in the Dlk1/Gtl2 imprinting region
Source: BMC Genomics. 2008 Jul 23;9:346. doi: 10.1186/1471-2164-9-346 (PMC2500034; doi:10.1186/1471-2164-9-346)
Supplement: Additional file 3 — Alignment of miRBase hsa-miR-154 family. [file 1471-2164-9-346-S3.pdf]

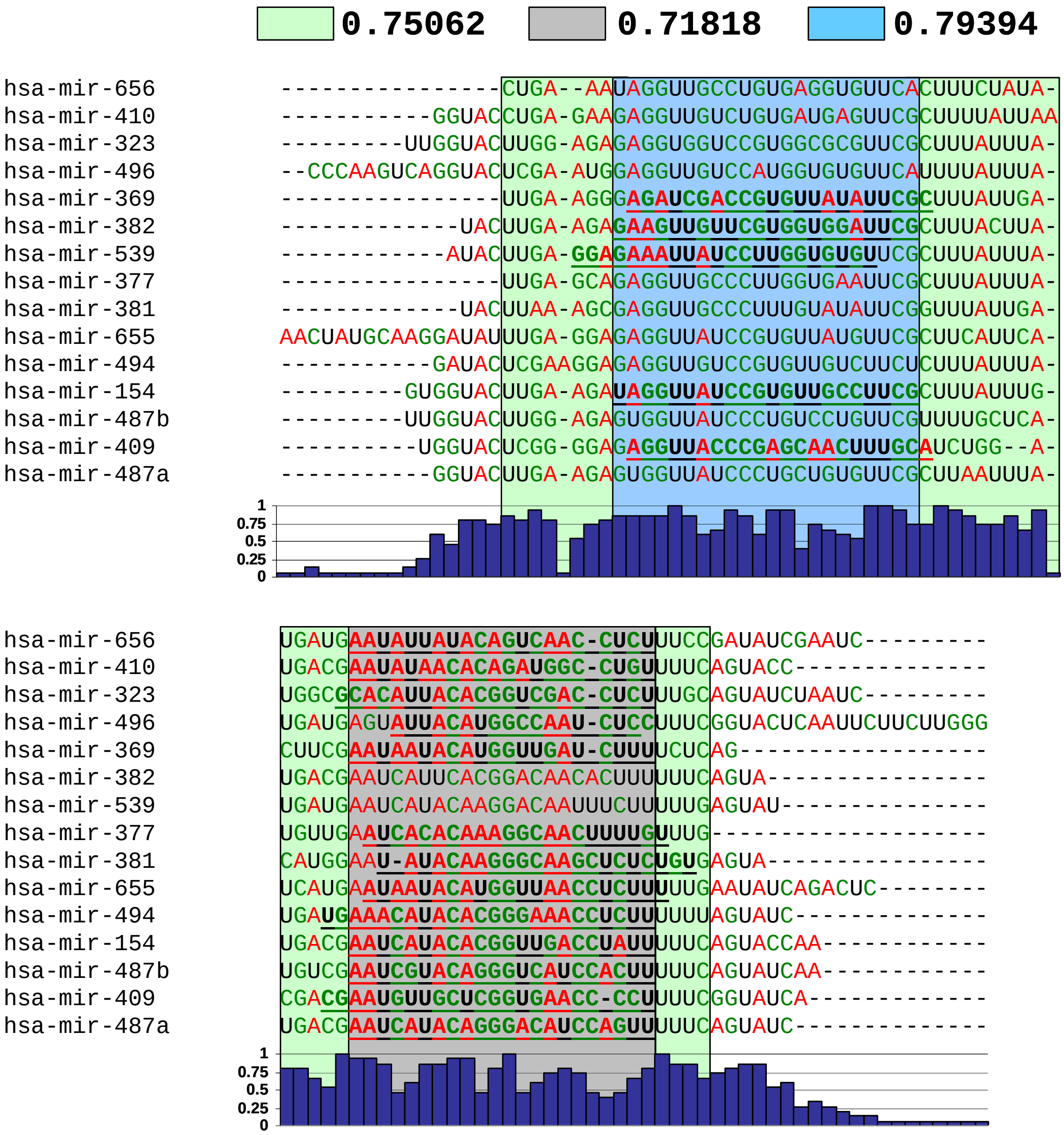

Figure S2: Multiple sequence alignment of the miRBase *hsa-miR-154* family shows small sequence variations, shifts in the hairpin structures causing different restriction sites, and differences in the selection of the mature microRNA exemplary for the known hairpin families. Shown is the conservation of each nucleotide within the family, including the 5' mature microRNA sequence (blue) as well as the 3' mature sequence of the *hsa-miR-154* (gray). Additionally, the average nucleotide conservation in each box is provided.
