# Supplementary material for: Structural conservation versus functional divergence of maternally expressed microRNAs in the Dlk1/Gtl2 imprinting region
Source: BMC Genomics. 2008 Jul 23;9:346. doi: 10.1186/1471-2164-9-346 (PMC2500034; doi:10.1186/1471-2164-9-346)
Supplement: Additional file 4 — Pair-wise mature sequence alignments. [file 1471-2164-9-346-S4.pdf]

**Supplemantary table S2:** Similar mature human and mouse microRNA sequences assigned using pairwise alignments with an SQ cutoff of 0.75. Non-orthologous pairs are highlighted in red.

| Human | Mouse | Sequence Alignment | Identity | Indel | *SQ* |
|---|---|---|---|---|---|
| hsa-miR-127 | mmu-miR-127 | UCGGAUCCGUCUGAGCUUGGCU<br>UCGGAUCCGUCUGAGCUUGGC-<br>******************** | 0.95 | 0.05 | **1.00** |
| hsa-miR-136 | mmu-miR-136 | ACUCCAUUUGUUUUGAUGAUGGA<br>ACUCCAUUUGUUUUGAUGAUGGA<br>*********************** | 1.00 | 0.00 | **1.00** |
| hsa-miR-337 | mmu-miR-337 | UCCAGCUCCUAUAUGAUGCCUUU<br>UUCAGCUCCUAUAUGAUGCCUUU<br>* ********************* | 0.96 | 0.00 | **0.95** |
| hsa-miR-370 | mmu-miR-370 | GCCUGCUGGGGUGGAACCUGG--<br>GCCUGCUGGGGUGGAACCUGGUU<br>********************* | 0.91 | 0.09 | **1.00** |
| hsa-miR-431 | mmu-miR-431 | UGUCUUGCAGGCCGUCAUGCA--<br>UGUCUUGCAGGCCGUCAUGCAGG<br>********************* | 0.91 | 0.09 | **1.00** |
| hsa-miR-433 | mmu-miR-433-3p | AUCAUGAUGGGCUCCUCGGUGU<br>AUCAUGAUGGGCUCCUCGGUGU<br>********************** | 1.00 | 0.00 | **1.00** |
| hsa-miR-134 | mmu-miR-134 | UGUGACUGGUUGACCAGAGGG-<br>UGUGACUGGUUGACCAGAGGGG<br>********************* | 0.95 | 0.05 | **1.00** |
| hsa-miR-154 | mmu-miR-154 | UAGGUUAUCCGUGUUGCCUUCG<br>UAGGUUAUCCGUGUUGCCUUCG<br>********************** | 1.00 | 0.00 | **1.00** |
| hsa-miR-154* | mmu-miR-485-3p | AAUCAUACACGGUUGACCUAUU<br>-GUCAUACACGGCUCUCCUCU-<br>  ********** *  *** * | 0.68 | 0.09 | **0.75** |
| | mmu-miR-369-3p | AAUCAUACACGGUUGACCUAUU<br>AAUAAUACAUGGUUGAUCUU--<br>*** ***** ****** ** | 0.73 | 0.09 | **0.80** |
| hsa-miR-299-5p | mmu-miR-299 | UGGUUUACCGUCCCACAUACAU<br>UGGUUUACCGUCCCACAUACAU<br>********************** | 1.00 | 0.00 | **1.00** |
| hsa-miR-323 | mmu-miR-323 | GCACAUUACACGGUCGACCUCU<br>GCACAUUACACGGUCGACCUCU<br>********************** | 1.00 | 0.00 | **1.00** |
| hsa-miR-329 | mmu-miR-329 | AACACACCUGGUUAACCUCUUU<br>AACACACCCAGCUAACCUUUUU<br>********  * ****** *** | 0.81 | 0.00 | **0.90** |
| hsa-miR-368 | mmu-miR-376c | -ACAUAGAGGAAAUUCCACGUUU<br>AACAUAGAGGAAAUUUCACG---<br> ************** **** | 0.78 | 0.17 | **0.90** |
| | mmu-miR-376a | -ACAUAGAGGAAAUUCCACGUUU<br>AUCGUAGAGGAAAAUCCACGU--<br>  * ********* ******* | 0.74 | 0.13 | **0.80** |
| | mmu-miR-376b | -ACAUAGAGGAAAUUCCACGUUU<br>AUCAUAGAGGAACAUCCACUUU-<br>  **********  ***** ** | 0.74 | 0.09 | **0.77** |
| hsa-miR-369-3p | mmu-miR-369-3p | AAUAAUACAUGGUUGAUCUUU<br>AAUAAUACAUGGUUGAUCUU-<br>******************** | 0.95 | 0.05 | **1.00** |
| hsa-miR-369-5p | mmu-miR-369-5p | AGAUCGACCGUGUUAUAUUCGC<br>AGAUCGACCGUGUUAUAUUCG-<br>********************* | 0.95 | 0.05 | **1.00** |
| hsa-miR-376a* | mmu-miR-376a* | GGUAGAUUCUCCUUCUAUGAG<br>GGUAGAUUCUCCUUCUAUGAG<br>********************* | 1.00 | 0.00 | **1.00** |

| Human | Mouse | Sequence Alignment | Identity | Indel | *SQ* |
|---|---|---|---|---|---|
| hsa-miR-376a | mmu-miR-376a | AUCAUAGAGGAAAAUCCACGU<br>AUCGUAGAGGAAAAUCCACGU<br>*** ***************** | 0.95 | 0.00 | **0.95** |
| | mmu-miR-376b | AUCAUAGAGGAAAAUCCACGU-<br>AUCAUAGAGGAACAUCCACUUU<br>************ ****** * | 0.86 | 0.05 | **0.90** |
| | mmu-miR-376c | AUCAUAGAGGAAAAUCCACGU<br>AACAUAGAGGAAAUUUCACG-<br>* *********** * **** | 0.81 | 0.05 | **0.85** |
| hsa-miR-376b | mmu-miR-376b | AUCAUAGAGGAAAAUCCAUGUU<br>AUCAUAGAGGAACAUCCACUUU<br>************ *****  ** | 0.86 | 0.00 | **0.86** |
| | mmu-miR-376a | AUCAUAGAGGAAAAUCCAUGUU<br>AUCGUAGAGGAAAAUCCACGU-<br>*** ************** ** | 0.86 | 0.05 | **0.90** |
| | mmu-miR-376c | AUCAUAGAGGAAAAUCCAUGUU<br>AACAUAGAGGAAAUUUCACG--<br>* *********** * ** * | 0.72 | 0.09 | **0.80** |
| hsa-miR-377 | mmu-miR-377 | AUCACACAAAGGCAACUUUUGU<br>AUCACACAAAGGCAACUUUUGU<br>********************** | 1.00 | 0.00 | **1.00** |
| hsa-miR-379 | mmu-miR-379 | UGGUAGACUAUGGAACGUA--<br>UGGUAGACUAUGGAACGUAGG<br>******************* | 0.90 | 0.10 | **1.00** |
| hsa-miR-380-3p | mmu-miR-380-3p | UAUGUAAUAUGGUCCACAUCUU<br>UAUGUAGUAUGGUCCACAUCUU<br>****** *************** | 0.95 | 0.00 | **0.95** |
| hsa-miR-380-5p | mmu-miR-380-5p | UGGUUGACCAUAGAACAUGCGC<br>UGGUUGACCAUAGAACAUGCGC<br>********************** | 1.00 | 0.00 | **1.00** |
| hsa-miR-381 | mmu-miR-381 | UAUACAAGGGCAAGCUCUCUGU<br>UAUACAAGGGCAAGCUCUCUGU<br>********************** | 1.00 | 0.00 | **1.00** |
| | mmu-miR-300 | UAUACAAGGGCAAGCUCUCUGU<br>UAUGCAAGGGCAAGCUCUCUUC<br>*** **************** | 0.86 | 0.00 | **0.86** |
| hsa-miR-382 | mmu-miR-382 | GAAGUUGUUCGUGGUGGAUUCG<br>GAAGUUGUUCGUGGUGGAUUCG<br>********************** | 1.00 | 0.00 | **1.00** |
| hsa-miR-409-3p | mmu-miR-409 | CGAAUGUUGCUCGGUGAACCCCU-<br>-GAAUGUUGCUCGGUGAACCCCUU<br> ********************** | 0.92 | 0.08 | **0.95** |
| hsa-miR-410 | mmu-miR-410 | AAUAUAACACAGAUGGCCUGU-<br>AAUAUAACACAGAUGGCCUGUU<br>********************* | 0.95 | 0.05 | **1.00** |
| hsa-miR-412 | mmu-miR-412 | ACUUCACCUGGUCCACUAGCCGU<br>ACUUCACCUGGUCCACUAGCCGU<br>*********************** | 1.00 | 0.00 | **1.00** |
| hsa-miR-453 | mmu-miR-154 | GAGGUUGUCCGUGGUGAGUUCG<br>UAGGUUAUCCGUGUUGCCUUCG<br> ***** ****** **  **** | 0.77 | 0.00 | **0.77** |
| | mmu-miR-485-5p | -GAGGUUGUCCGUGGUGAGUUCG<br>AGAGGCUGGCCGUGAUGAAUUC-<br> **** ** ***** *** *** | 0.74 | 0.09 | **0.77** |
| | mmu-miR-382 | GAGGUUGUCCGUGGUGAGUUCG<br>GAAGUUGUUCGUGGUGGAUUCG<br>** ***** *******  **** | 0.82 | 0.00 | **0.81** |
| hsa-miR-485-3p | mmu-miR-485-3p | GUCAUACACGGCUCUCCUCUCU<br>GUCAUACACGGCUCUCCUCU--<br>******************** | 0.91 | 0.09 | **1.00** |

| Human | Mouse | Sequence Alignment | Identity | Indel | *SQ* |
|---|---|---|---|---|---|
| hsa-miR-485-5p | mmu-miR-485-5p | AGAGGCUGGCCGUGAUGAAUUC<br>AGAGGCUGGCCGUGAUGAAUUC<br>********************** | 1.00 | 0.00 | **1.00** |
| hsa-miR-487a | mmu-miR-487b | AAUCAUACAGGGACAUCCAGUU<br>AAUCGUACAGGGUCAUCCACU-<br>**** ******* ****** * | 0.82 | 0.05 | **0.85** |
| | mmu-miR-376b | AAUCAUACAGGGACAUCCAGUU-<br>-AUCAUAGAGGAACAUCCACUUU<br> ****** *** ******* ** | 0.77 | 0.09 | **0.81** |
| hsa-miR-487b | mmu-miR-487b | AAUCGUACAGGGUCAUCCACUU<br>AAUCGUACAGGGUCAUCCACU-<br>********************* | 0.95 | 0.05 | **1.00** |
| | mmu-miR-376a | AAUCGUACAGGGUCAUCCACUU<br>-AUCGUAGAGGAAAAUCCACGU<br> ****** ***   ****** * | 0.73 | 0.05 | **0.76** |
| | mmu-miR-376b | AAUCGUACAGGGUCAUCCACUU-<br>-AUCAUAGAGGAACAUCCACUUU<br> *** ** ***  ********* | 0.74 | 0.09 | **0.77** |
| hsa-miR-494 | mmu-miR-494 | UGAAACAUACACGGGAAACCUCUU<br>UGAAACAUACACGGGAAACCU---<br>********************* | 0.88 | 0.13 | **1.00** |
| hsa-miR-495 | mmu-miR-495 | AAACAAACAUGGUGCACUUCUUU<br>AAACAAACAUGGUGCACUUCUU-<br>********************** | 0.96 | 0.04 | **1.00** |
| | mmu-miR-543 | AAACAAACAUGGUGCACUUCUUU<br>AAACAUUCGCGGUGCACUUCU--<br>*****  *  *********** | 0.74 | 0.09 | **0.80** |
| hsa-miR-496 | mmu-miR-323 | ----AUUACAUGGCCAAUCUC-<br>GCACAUUACACGGUCGACCUCU<br>    ****** ** * * *** | 0.59 | 0.23 | **0.76** |
| hsa-miR-539 | mmu-miR-539 | GGAGAAAUUAUCCUUGGUGUGU<br>GGAGAAAUUAUCCUUGGUGUGU<br>********************** | 1.00 | 0.00 | **1.00** |
| hsa-miR-654 | mmu-miR-380-5p | UGGUGGGCCGCAGAACAUGUGC<br>UGGUUGACCAUAGAACAUGCGC<br>**** * **  ******** ** | 0.77 | 0.00 | **0.77** |
| hsa-miR-655 | mmu-miR-369-3p | -AUAAUACAUGGUUAACCUCUUU<br>AAUAAUACAUGGUUGAUCUU---<br> ************* * ** | 0.70 | 0.17 | **0.80** |
| hsa-miR-656 | mmu-miR-323 | -AAUAUUAUACAGUCAACCUCU<br>GCACAUUACACGGUCGACCUCU<br>  * **** ** *** ****** | 0.73 | 0.05 | **0.76** |
