# Supplementary material for: Structural conservation versus functional divergence of maternally expressed microRNAs in the Dlk1/Gtl2 imprinting region
Source: BMC Genomics. 2008 Jul 23;9:346. doi: 10.1186/1471-2164-9-346 (PMC2500034; doi:10.1186/1471-2164-9-346)
Supplement: Additional file 5 — Detailed description of target prediction approach and validation. [file 1471-2164-9-346-S5.pdf]

Supplementary text: Predicting microRNA target sites using a combined target gene prediction approach

Martin Kircher[1,3], Christoph Bock[1], Martina Paulsen[2]

[1] Max-Planck-Institut für Informatik, Saarbrücken, Germany
[2] Genetik/Epigenetik, Universität des Saarlandes, Saarbrücken, Germany
[3] Present address: Max-Planck-Institut für Evolutionäre Anthropologie, Leipzig, Germany

Contents

**1**	**Summary**	**1**
**2**	**Outline of a combined microRNA target gene prediction approach**	**1**
**3**	**Implementation details – prediction algorithms**	**2**
**4**	**Implementation details – combination strategy**	**2**
**5**	**Implementation details – filtering strategies**	**3**
**6**	**Target validation**	**3**
**Tables**		**5**

1 Summary

MicroRNA target prediction is a complex task with many free parameters. In this Supplementary document, we describe the combined target gene prediction approach used for the bioinformatic analysis of post-transcriptional gene regulation by the microRNAs in the *Dlk1/Gtl2* imprinting region.

2 Outline of a combined microRNA target gene prediction approach

A variety of strategies has been proposed for computationally predicting microRNA target genes (see [1-4] for review). One important methodology uses complete annealing of microRNA and mRNA (similar to local alignments), as in miRanda [5], DIANA-microT [6], RNAhybrid [7] and the algorithm by Watanabe et al. [8]. Another methodology uses string matching of the seed region (potentially with an extension to a full alignment) as in microInspector [9], PicTar [10], TargetScan [11], TargetScanS [12] and the algorithms by Stark et al. [13]/Brennecke et al. [14]. These algorithms use specific parameters for filtering their predicted targets, such as score value cutoffs, experimental information coupled with empirical rules (e.g. number of G:U wobbles and the predicted bulge position), or annotation data (e.g. target function and tissue specificity). Therefore, any overlap observed between the predictions of different programs is relatively small [2, 4, 15].

It is a common observation from multiple areas in bioinformatics, including protein structure prediction, protein function prediction and gene prediction, that consensus methods can achieve higher prediction accuracy and robustness than any of the underlying algorithms alone [16]. Therefore, we decided to exploit the diversity of available methods for microRNA target prediction by using a combination of different prediction algorithms. We included three widely used and complementary prediction strategies:

- The miRanda algorithm [5], which is based on a dynamic programming alignment of microRNA and mRNA sequences, with subsequent calculation of the microRNA:target duplex binding energy using RNA folding algorithms and filtering based on empirical binding rules.

- The RNAhybrid algorithm [7], which uses short nucleic acid hybridizations and estimated p-values to predict binding sites without the need for filtering by empirical binding rules.

- Our custom SeedMatch algorithm, which uses basic string matching of the microRNA seed region, highly similar to TargetScanS [12] and PicTar [10]. Briefly, this method searches for the classical perfect 5’ seed match to at least six bases, but also considers 3’ compensation for minimal 5’ pairing [14] by searching for a perfect 3’ seed. If both seeds are available and their extensions do not conflict, the length sum is used for scoring; otherwise, only the length of the 5’ seed is used.

To combine the score values from the different methods, we used unweighted majority voting based on the predictions of complete transcripts, as illustrated in figure 3. This combination strategy is more suitable than taking the union or intersection of predicted targets [4], since the number of significant target sites reported by the different algorithms varies widely. However, it requires us to introduce new thresholds into the consensus prediction (see below).

3 Implementation details – prediction algorithms

Each of the three prediction algorithms was applied separately to predict microRNA target genes in the sets of mouse and human 3’ UTR sequences and repetitive elements, using the following settings:

- **miRanda algorithm:** miRanda [5] 1.0b for Linux was used for prediction. Except for the energy cutoff for the microRNA:mRNA duplex – which was set to -15 kcal/mol for higher specificity of the antitargets – the default parameters (alignment score threshold = 50.0, scale parameter = 2.0, usage of strict duplex heuristics) were used.

- **RNAhybrid algorithm:** RNAhybrid [7] 2.1 was used with the parameters “-s 3utr_human” to base the p-value calculation on an estimate of human 3’ UTR base composition and “-e 15” to set the threshold on the microRNA:target duplex energy to -15 kcal/mol. To supplement the energy and p-value, an alignment score was calculated from the RNAhybrid output by scoring base pairings of G:U with 1, A:U with 2 and G:C with 3 and to score gaps with a score of -1 for extension and -2 for opening (gap costs are averaged for both sides of the duplex alignment).

- **SeedMatch algorithm:** In the first step of the algorithm, the bases 2-6 (begin 5’, referred to as seed) and 2-5 (begin 3’, referred to as 3’ seed) of the microRNA sequence were extracted and reverse complemented. For each given sequence, all matches to the seed and 3’ seed matches of a distance of 3 to 23 bases were identified. For each seed found, the matching was extended in both directions iteratively. After the extension step, all seed matches smaller than six bases were rejected. For the remaining seeds, the corresponding 3’ seed matches were extended. After this step, only the longest 3’ seed was saved. If there was an overlap of 3’ seed and seed, the 3’ seed was rejected. Finally for a given sequence, the algorithm returns a list of seed matches with a length of at least six bases and, if available, a corresponding 3’ seed match. For our analysis, only the length of each seed and the 3’ seed were considered. Their sum was used as the score.

These three methods produce six scores: the miRanda energy score, the miRanda alignment score, the RNAhybrid estimated p-value, the RNAhybrid energy score, the RNAhybrid derived alignment score and the SeedMatch length. All scores were treated as independent inputs for the consensus algorithm, due to the relatively low correlation between different scores produced by the same algorithm (Pearson’s $R^2 < 0.5$ in all cases).

4 Implementation details – combination strategy

The binding sites scores predicted in each of the inputs were filtered, and the remaining scores were combined to obtain only one score for each target sequence and input. For RNAhybrid p-values, the product of all remaining binding site scores, and the square root of a residual sum of squares was calculated, since this seems to best model the behavior observed for multiple target sites [3, 5]. Hence, a large cutoff difference received a high score, which may have also been achieved using multiple sites with a low cutoff difference.

After combining several binding site scores with a sequence score, the resulting scores were adjusted to guarantee an equal value range for the different inputs. In the score adjustment, the 25th and the 75th percentiles of the sequence scores from one input over all mature microRNA sequences (in one species) were used for a linear scaling. The value of the 25th percentile was set to -1, and the value of the 75th percentile to 1. For RNAhybrid p-values, the quantiles were interchanged to obtain a positive orientation of this score.

Afterwards, the overlap between the resulting sets of predicted sequences obtained from the different inputs was evaluated. If a sequence was predicted in at least n of the six inputs, the average of the supporting scores was calculated, and both the sequence identifier and average score were appended to the final list of predicted sequences. Using a mapping of mouse and human sequence identifiers, the final results for mouse and human were filtered for target sequences conserved between the species. By not enforcing conservation of individual sites, we account for binding sites that disappear and evolve over the sequence. We set the parameter n to four when we used the conservation filter and to five otherwise.

We defined antitargets as all sequence identifiers not predicted in the raw prediction data of a specific microRNA sequence with any of the three prediction algorithms. For each species and microRNA sequence, we therefore extracted the sequence identifiers not predicted by miRanda, RNAhybrid and SeedMatch and then calculated their intersection. By this definition, antitargets are independent of any defined binding site filter. As for the targets, we filtered the final results for sequences predicted in mouse and human to obtain conserved antitarget sequence identifiers.

5 Implementation details – filtering strategies

For the filter step, in which all binding sites smaller or equal to a cutoff were excluded, three different strategies were developed and verified:

Classic This filter assumes that a separation between functional and non-functional binding sites for each score is independent of a specific microRNA sequence. Therefore, Classic defines the cutoff values irrespective of the individual microRNA and uses the fifth percentile of each score predicted over all mature microRNA sequences in the *Dlk1/Gtl2* region in one species. The fifth percentile is an arbitrary choice, resulting in proper values (table 2). Common to all filter strategies, the 25th and the 75th percentiles of combined sequence scores were used for equal scaling (table 3).

Vari The second filter assumes a separation between functional and non-functional binding sites relative to a maximum score for the specific microRNA sequence. It uses the alignment and energy scores determined for each individual mature microRNA and its reverse complement as a target sequence (for calculation, miRanda was used without the strict duplex heuristics). We extracted the scores given in table 4 (*Dlk1/Gtl2*) and table 5 (reference set), and set the cutoff to 60% of the determined scores. For the remaining scores (p-value and SeedMatch length), the same cutoff values as in the Classic filter were used (table 2). The 25th and the 75th percentiles are provided in table 6.

3000 The last filter assumes that each mature microRNA sequence is available in a fixed copy number (assuming equal transcription and processing of the microRNAs in the clusters) and will interact with its best 3000 predicted target sequences. To exclude very low scores, we restricted the different inputs by the cutoffs reported in table 7, before extracting the 3000 best scoring sequence identifiers of each predictor. The determined 25th and 75th percentiles are given in table 8.

For the resulting combined predictions, the constraints of the 3000 and Vari strategies were observed to be tighter than for Classic (figure 1). Therefore, the 3000 and Vari targets are subsets of the Classic targets, apart from a few outliers. Even though the filter strategies follow different assumptions and have different target sets, they still showed an overlap in over 50% of the results of the smallest filter strategy.

6 Target validation

It is important to assess experimentally the sensitivity and selectivity of each prediction approach. Since we do not have our own experimental data, we used a set of 129 *mmu-mir-134* targets verified with Luciferase-reporter assays by Miranda et al. [17] and tested their recovery rate. Of their 129 Ensembl Transcript IDs, 101 were included in our sequence set used for prediction. A subset of 87 transcripts could be mapped to a human sequence identifier for conservation filtering. We calculated the enrichment E with equation (1) and the probability p for a hypergeometric distribution with equation (2). The parameters were the number of observations (predicted targets) n, the number of all sequence identifiers N, the number of verified identifiers D and the number of verified predicted targets k.

$$E = \frac{k \cdot N}{D \cdot n} \qquad (1)$$

$$p = \frac{\binom{D}{k}\binom{N-D}{n-k}}{\binom{N}{n}} \qquad (2)$$

All target sets, except the conserved 3000, were significantly enriched (significance cutoff of 0.01, table 1); none retrieved all targets, but Vari performed the best with respect to enrichment and hypergeometric probability.

Table 1: Enrichment values and hypergeometric probabilities of the validated *mmu-mir-134* target set for the three filter strategies. The label "con." is short for conserved and refers to predicted mouse targets that passed the conservation filter.

	Targets	Validated	Ratio validated	Enrichment	Probability
Classic	2681	61	0.60396	4.56383	1.27085E-28
Vari	489	26	0.25743	10.66501	7.61751E-20
3000	554	24	0.23762	8.68957	2.52511E-16
con. Classic	1059	18	0.20690	2.72619	5.45773E-05
con. Vari	207	9	0.10345	6.97351	4.90060E-06
con. 3000	98	1	0.01149	1.63664	3.34727E-01

Targets of the Dlk1/Gtl2 region

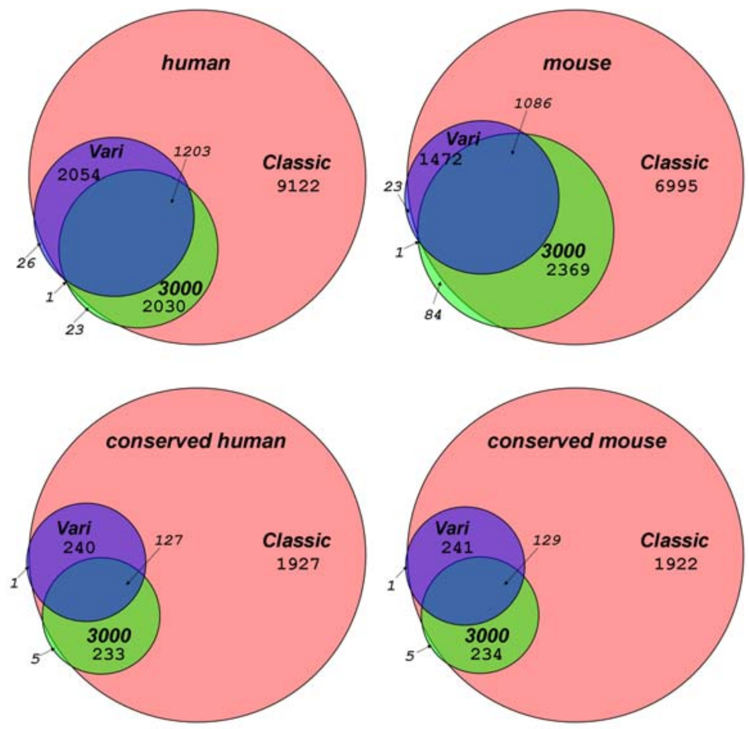

Targets of randomly selected microRNAs (reference set)

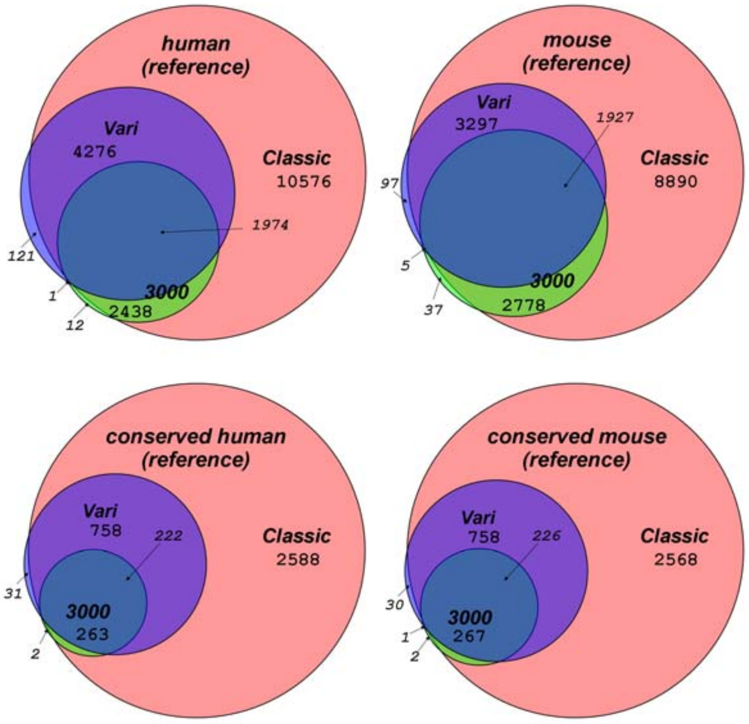

Figure 1: Venn diagrams of target sequences predicted for more than two of the *Dlk1/Gtl2* microRNAs and more than two of the randomly selected microRNAs (reference set). The disk sizes are relative to the largest of the three target sets in each of the eight Venn diagrams. "Conserved" refers to target sequences predicted for both species (small differences in these numbers are due to target identifiers with identical RefSeq/EMBL sequence identifiers). The difference between the human and mouse results in both microRNA sets is presumably caused by the larger number of annotated 3' UTR sequences, two additional mature sequences and the higher number of microRNA sequence similarities observed in human. Even though the filter strategies follow different assumptions and have different target sets, they still showed an overlap in over 50% of the smallest result.

Tables

Table 2: Fifth percentile of *Dlk1/Gtl2* scores used as the Classic cutoff for the *Dlk1/Gtl2* microRNAs and the reference set. Since SeedMatch predicts about five times fewer sites than miRanda, while RNAhybrid predicts about five times more sites than miRanda, the SeedMatch length cutoff was reduced to greater than or equal to eight. For the actual implementation, we used the value 7.98, because the remaining binding sites were defined to be larger than the cutoff. In percentiles, this value corresponds to the thirteenth percentile in mouse and human for the *Dlk1/Gtl2* microRNAs.

Score	5th (Mouse)	5th (Human)
miRanda energy score	-22.79	-23.19
miRanda alignment score	97.0	96.0
RNAhybrid energy score	-23.70	-23.90
RNAhybrid estimated p-value	0.71886	0.69580
RNAhybrid derived alignment score	29.00	28.50
SeedMatch length	*10*	*10*

Table 3: 25th and 75th percentile of combined sequence scores used for equal scaling of the scores for the *Dlk1/Gtl2* microRNAs and the reference set (Classic filter).

Score	*Dlk1/Gtl2*				Reference set			
	Mouse		Human		Mouse		Human	
	25th	75th	25th	75th	25th	75th	25th	75th
miRanda energy score	0.74	3.52	0.80	3.63	0.90	4.27	0.81	3.69
miRanda alignment score	3.00	10.00	3.00	10.00	3.00	10.00	3.00	11.00
RNAhybrid energy score	0.94	4.19	1.00	4.50	1.20	5.54	1.10	4.80
RNAhybrid estimated p-value	0.30477	0.72723	0.23511	0.69888	0.23331	0.70403	0.24318	0.69587
RNAhybrid derived alignment score	1.50	5.02	1.50	5.50	1.58	6.20	1.58	6.02
SeedMatch length	0.02	3.02	0.02	3.02	0.02	3.02	0.02	3.02

Table 4: miRanda and RNAhybrid scores for each microRNA of the *Dlk1/Gtl2* region with its inverse as target (Vari filter).

microRNA	miRanda energy	miRanda alignment	RNAhybrid energy	RNAhybrid alignment
hsa-miR-127	-49.07	165.0	-46.1	50.5
hsa-miR-134	-46.05	160.0	-41.8	46.5
hsa-miR-136	-38.9	170.0	-37.0	47.5
hsa-miR-154	-44.09	165.0	-41.1	47.5
hsa-miR-154*	-38.31	165.0	-38.4	45.5
hsa-miR-299-3p	-42.46	165.0	-42.2	47.5
hsa-miR-299-5p	-43.13	165.0	-41.1	47.5
hsa-miR-323	-46.59	165.0	-41.7	48.5
hsa-miR-329	-39.88	165.0	-40.5	46.5
hsa-miR-337	-43.86	170.0	-43.4	49.5
hsa-miR-368	-37.87	165.0	-36.9	45.5
hsa-miR-369-3p	-30.08	160.0	-31.2	40.5
hsa-miR-369-5p	-41.56	165.0	-36.9	46.5
hsa-miR-370	-50.87	160.0	-46.1	48.5
hsa-miR-376a	-37.27	160.0	-36.2	43.5
hsa-miR-376a*	-39.46	160.0	-34.7	42.5
hsa-miR-376b	-36.19	165.0	-36.8	44.5
hsa-miR-377	-36.53	165.0	-36.6	45.5
hsa-miR-379	-35.34	150.0	-33.7	39.5
hsa-miR-380-3p	-37.0	165.0	-36.9	44.5
hsa-miR-380-5p	-43.21	165.0	-39.8	47.5
hsa-miR-381	-42.38	165.0	-42.1	47.5
hsa-miR-382	-43.7	165.0	-38.8	46.5
hsa-miR-409-3p	-50.12	170.0	-45.5	51.5
hsa-miR-409-5p	-43.04	165.0	-41.6	48.5
hsa-miR-410	-36.69	160.0	-37.0	43.5
hsa-miR-411	-41.55	160.0	-38.0	44.5
hsa-miR-412	-50.43	170.0	-48.3	52.5
hsa-miR-431	-44.28	160.0	-43.0	47.5
hsa-miR-432	-48.64	170.0	-43.9	50.5
hsa-miR-432s	-46.53	160.0	-43.5	46.5

microRNA	miRanda energy	miRanda alignment	RNAhybrid energy	RNAhybrid alignment
hsa-miR-433	-47.32	165.0	-46.4	49.5
hsa-miR-453	-48.62	165.0	-43.5	48.5
hsa-miR-485-3p	-47.37	165.0	-44.2	48.5
hsa-miR-485-5p	-45.36	165.0	-43.6	48.5
hsa-miR-487a	-40.89	165.0	-41.5	46.5
hsa-miR-487b	-42.98	165.0	-42.9	47.5
hsa-miR-494	-45.16	175.0	-43.0	51.5
hsa-miR-495	-37.54	170.0	-38.8	47.5
hsa-miR-496	-28.53	140.0	-29.0	33.5
hsa-miR-539	-40.28	165.0	-36.3	45.5
hsa-miR-544	-29.29	155.0	-30.4	39.5
hsa-miR-654	-49.2	165.0	-46.2	50.5
hsa-miR-655	-33.66	165.0	-34.3	43.5
hsa-miR-656	-32.92	160.0	-32.6	41.5
mmu-miR-127	-46.95	160.0	-42.7	47.5
mmu-miR-134	-49.57	165.0	-45.1	49.5
mmu-miR-136	-38.9	170.0	-37.0	47.5
mmu-miR-154	-44.09	165.0	-41.1	47.5
mmu-miR-299	-43.13	165.0	-41.1	47.5
mmu-miR-300	-42.85	165.0	-42.8	47.5
mmu-miR-323	-46.59	165.0	-41.7	48.5
mmu-miR-329	-38.93	165.0	-40.2	46.5
mmu-miR-337	-41.37	170.0	-42.6	48.5
mmu-miR-341	-49.12	160.0	-44.0	48.5
mmu-miR-369-3p	-29.05	155.0	-30.3	38.5
mmu-miR-369-5p	-38.98	160.0	-34.6	43.5
mmu-miR-370	-53.74	170.0	-51.6	53.5
mmu-miR-376a	-39.36	160.0	-37.6	44.5
mmu-miR-376a*	-39.46	160.0	-34.7	42.5
mmu-miR-376b	-38.62	165.0	-39.1	45.5
mmu-miR-376b*	-37.27	160.0	-35.9	42.5
mmu-miR-376c	-32.51	155.0	-31.0	39.5
mmu-miR-377	-36.53	165.0	-36.6	45.5
mmu-miR-379	-41.98	160.0	-37.2	44.5
mmu-miR-380-3p	-39.43	165.0	-39.2	45.5
mmu-miR-380-5p	-43.21	165.0	-39.8	47.5
mmu-miR-381	-42.38	165.0	-42.1	47.5
mmu-miR-382	-43.7	165.0	-38.8	46.5
mmu-miR-409	-47.29	170.0	-45.4	50.5
mmu-miR-410	-37.7	165.0	-39.0	45.5
mmu-miR-411	-48.54	170.0	-47.0	51.5
mmu-miR-412	-50.43	170.0	-48.3	52.5
mmu-miR-431	-50.92	170.0	-47.5	52.5
mmu-miR-433-3p	-47.32	165.0	-46.4	49.5
mmu-miR-433-5p	-42.01	165.0	-40.2	46.5
mmu-miR-434-3p	-41.02	160.0	-37.9	44.5
mmu-miR-434-5p	-43.41	165.0	-39.8	47.5
mmu-miR-485-3p	-42.47	155.0	-39.7	43.5
mmu-miR-485-5p	-45.36	165.0	-43.6	48.5
mmu-miR-487b	-41.89	160.0	-40.7	45.5
mmu-miR-494	-39.17	160.0	-36.3	44.5
mmu-miR-495	-36.51	165.0	-37.9	45.5
mmu-miR-539	-40.28	165.0	-36.3	45.5
mmu-miR-540	-45.0	155.0	-40.8	44.5
mmu-miR-541	-43.82	170.0	-43.0	49.5
mmu-miR-543	-39.47	160.0	-39.0	45.5
mmu-miR-679	-47.51	165.0	-43.2	48.5

Table 5: miRanda and RNAhybrid scores for each microRNA of the reference set with its inverse as target (Vari filter).

microRNA	miRanda energy	miRanda alignment	RNAhybrid energy	RNAhybrid alignment
hsa-let-7f	-37.22	165.0	-36.0	44.5
hsa-miR-103	-44.66	170.0	-44.0	50.5
hsa-miR-107	-44.66	170.0	-44.2	50.5
hsa-miR-10a	-43.98	170.0	-41.2	48.5
hsa-miR-132	-44.56	165.0	-42.1	47.5

microRNA	miRanda energy	miRanda alignment	RNAhybrid energy	RNAhybrid alignment
hsa-miR-135b	-37.42	165.0	-36.2	44.5
hsa-miR-141	-40.39	165.0	-37.2	45.5
hsa-miR-143	-42.31	165.0	-40.9	47.5
hsa-miR-155	-40.04	165.0	-37.3	45.5
hsa-miR-15b	-39.56	165.0	-39.3	46.5
hsa-miR-17-3p	-38.79	155.0	-38.6	43.5
hsa-miR-17-5p	-46.42	175.0	-43.9	51.5
hsa-miR-181a	-45.18	170.0	-43.8	50.5
hsa-miR-181a*	-44.11	165.0	-39.5	47.5
hsa-miR-181d	-44.35	175.0	-44.1	51.5
hsa-miR-185	-34.51	145.0	-33.0	37.5
hsa-miR-186	-40.51	170.0	-39.0	47.5
hsa-miR-194	-42.34	165.0	-40.2	47.5
hsa-miR-195	-36.93	160.0	-36.0	43.5
hsa-miR-205	-47.37	165.0	-43.4	48.5
hsa-miR-215	-36.57	160.0	-35.7	42.5
hsa-miR-22	-41.07	165.0	-41.7	47.5
hsa-miR-220	-41.84	160.0	-38.0	44.5
hsa-miR-25	-44.58	165.0	-40.9	47.5
hsa-miR-26a	-38.57	160.0	-36.8	43.5
hsa-miR-301	-39.71	170.0	-36.0	46.5
hsa-miR-324-3p	-54.31	165.0	-49.4	51.5
hsa-miR-342	-50.9	175.0	-46.8	53.5
hsa-miR-361	-43.04	165.0	-41.6	46.5
hsa-miR-424	-36.21	165.0	-35.7	44.5
hsa-miR-429	-38.51	165.0	-36.6	45.5
hsa-miR-486	-50.99	165.0	-47.8	50.5
hsa-miR-490	-49.02	165.0	-45.5	48.5
hsa-miR-513	-40.95	165.0	-41.0	46.5
hsa-miR-518a	-41.03	160.0	-41.5	46.5
hsa-miR-525	-43.85	160.0	-40.9	45.5
hsa-miR-545	-34.06	165.0	-33.6	43.5
hsa-miR-551a	-44.76	160.0	-40.7	46.5
hsa-miR-555	-41.31	160.0	-41.1	45.5
hsa-miR-556	-32.66	155.0	-30.4	38.5
hsa-miR-578	-39.2	160.0	-36.5	43.5
hsa-miR-580	-36.04	165.0	-33.5	43.5
hsa-miR-589	-49.01	175.0	-45.7	53.5
hsa-miR-601	-45.34	165.0	-42.0	47.5
hsa-miR-662	-48.92	160.0	-46.0	48.5
mmu-let-7f	-36.21	160.0	-34.0	42.5
mmu-miR-101a	-38.5	165.0	-36.3	41.0
mmu-miR-103	-44.66	170.0	-44.0	50.5
mmu-miR-107	-44.66	170.0	-44.2	50.5
mmu-miR-10a	-43.98	170.0	-41.2	48.5
mmu-miR-132	-44.56	165.0	-42.1	47.5
mmu-miR-135b	-37.42	165.0	-36.2	44.5
mmu-miR-141	-40.39	165.0	-37.2	45.5
mmu-miR-143	-42.31	165.0	-40.9	47.5
mmu-miR-155	-37.54	165.0	-35.5	44.5
mmu-miR-15b	-39.56	165.0	-39.3	46.5
mmu-miR-17-3p	-42.48	160.0	-42.9	46.5
mmu-miR-17-5p	-46.42	175.0	-43.9	51.5
mmu-miR-181a	-45.18	170.0	-43.8	50.5
mmu-miR-181a*	-44.11	165.0	-39.5	47.5
mmu-miR-185	-34.51	145.0	-33.0	37.5
mmu-miR-186	-40.51	170.0	-39.0	47.5
mmu-miR-194	-42.34	165.0	-40.2	47.5
mmu-miR-195	-36.93	160.0	-36.0	43.5
mmu-miR-201	-36.89	160.0	-36.2	43.5
mmu-miR-205	-47.37	165.0	-43.4	48.5
mmu-miR-215	-36.57	160.0	-35.7	42.5
mmu-miR-22	-41.07	165.0	-41.7	47.5
mmu-miR-25	-44.58	165.0	-40.9	47.5
mmu-miR-26a	-38.57	160.0	-36.8	43.5
mmu-miR-301	-39.71	170.0	-36.0	46.5
mmu-miR-324-3p	-54.31	165.0	-49.4	51.5
mmu-miR-342	-50.9	175.0	-46.8	53.5

microRNA	miRanda energy	miRanda alignment	RNAhybrid energy	RNAhybrid alignment
mmu-miR-351	-55.5	175.0	-52.8	55.5
mmu-miR-361	-43.04	165.0	-41.6	46.5
mmu-miR-424	-38.7	165.0	-36.5	45.5
mmu-miR-429	-40.59	165.0	-39.2	46.5
mmu-miR-486	-50.99	165.0	-47.8	50.5
mmu-miR-490	-49.02	165.0	-45.5	48.5
mmu-miR-546	-34.04	135.0	-33.8	34.5
mmu-miR-670	-43.04	165.0	-43.5	47.5
mmu-miR-675-5p	-50.65	165.0	-48.6	51.5
mmu-miR-677	-38.21	165.0	-37.6	45.5
mmu-miR-680	-48.98	160.0	-44.2	47.5
mmu-miR-688	-38.82	155.0	-36.1	42.5
mmu-miR-689	-57.95	160.0	-51.1	51.5
mmu-miR-700	-51.55	160.0	-45.2	48.5
mmu-miR-717	-44.16	165.0	-40.4	46.5

Table 6: 25th and 75th percentile of combined sequence scores used for equal scaling of the scores for the *Dlk1/Gtl2* microRNAs and the reference set (Vari filter).

Score	*Dlk1/Gtl2*				Reference set			
	Mouse		Human		Mouse		Human	
	25th	75th	25th	75th	25th	75th	25th	75th
miRanda energy score	0.42	1.98	0.44	2.07	0.43	2.05	0.47	2.13
miRanda alignment score	2.00	9.00	3.00	9.00	3.0	10.0	3.0	10.0
RNAhybrid energy score	0.58	2.58	0.64	2.76	0.76	3.20	0.80	3.38
RNAhybrid estimated p-value	0.30477	0.72723	0.23511	0.69888	0.23331	0.70403	0.24318	0.69587
RNAhybrid derived alignment score	1.30	4.73	1.22	4.72	1.70	6.04	1.60	5.85
SeedMatch length	0.02	3.02	0.02	3.02	0.02	3.02	0.02	3.02

Table 7: Pre-filter cutoffs used in strategy 3000 for the *Dlk1/Gtl2* microRNAs and the reference set.

Score	Mouse	Human
miRanda energy score	-20.00	-20.00
miRanda alignment score	75.00	75.00
RNAhybrid energy score	-20.0	-20.00
RNAhybrid estimated p-value	1.00	1.00
RNAhybrid derived alignment score	15.00	15.00
SeedMatch length	5.95	5.95

Table 8: 25th and 75th percentile of combined sequence scores used for equal scaling of the scores for the *Dlk1/Gtl2* microRNAs and the reference set (3000 filter).

Score	*Dlk1/Gtl2*				Reference set			
	Mouse		Human		Mouse		Human	
	25th	75th	25th	75th	25th	75th	25th	75th
miRanda energy score	1.77	6.00	2.25	7.64	1.63	6.64	2.06	6.67
miRanda alignment score	25.00	37.39	26.00	37.70	28.50	39.00	29.27	41.07
RNAhybrid energy score	5.42	12.96	4.22	15.59	6.83	16.51	6.77	17.27
RNAhybrid estimated p-value	0.11799	0.51875	0.05686	0.60283	0.06827	0.40642	0.05148	0.40795
RNAhybrid derived alignment score	33.38	52.80	29.25	57.47	41.58	62.11	42.62	63.28
SeedMatch length	1.05	4.05	1.05	5.05	1.05	4.05	1.49	5.05

References

1. Bentwich I: **Prediction and validation of microRNAs and their targets**. *FEBS letters* 2005, **579**(26):5904-5910.
2. Rajewsky N: **microRNA target predictions in animals**. *Nature genetics* 2006, **38 Suppl**:S8-13.

3. Sachidanandam R: **RNAi as a bioinformatics consumer**. *Briefings in bioinformatics* 2005, **6**(2):146-162.
4. Sethupathy P, Megraw M, Hatzigeorgiou AG: **A guide through present computational approaches for the identification of mammalian microRNA targets**. *Nature methods* 2006, **3**(11):881-886.
5. Enright AJ, John B, Gaul U, Tuschl T, Sander C, Marks DS: **MicroRNA targets in Drosophila**. *Genome biology* 2003, **5**(1):R1.
6. Kiriakidou M, Nelson PT, Kouranov A, Fitziev P, Bouyioukos C, Mourelatos Z, Hatzigeorgiou A: **A combined computational-experimental approach predicts human microRNA targets**. *Genes & development* 2004, **18**(10):1165-1178.
7. Rehmsmeier M, Steffen P, Hochsmann M, Giegerich R: **Fast and effective prediction of microRNA/target duplexes**. *RNA (New York, NY* 2004, **10**(10):1507-1517.
8. Watanabe Y, Yachie N, Numata K, Saito R, Kanai A, Tomita M: **Computational analysis of microRNA targets in Caenorhabditis elegans**. *Gene* 2006, **365**:2-10.
9. Rusinov V, Baev V, Minkov IN, Tabler M: **MicroInspector: a web tool for detection of miRNA binding sites in an RNA sequence**. *Nucleic acids research* 2005, **33**(Web Server issue):W696-700.
10. Krek A, Grun D, Poy MN, Wolf R, Rosenberg L, Epstein EJ, MacMenamin P, da Piedade I, Gunsalus KC, Stoffel M *et al*: **Combinatorial microRNA target predictions**. *Nature genetics* 2005, **37**(5):495-500.
11. Lewis BP, Shih IH, Jones-Rhoades MW, Bartel DP, Burge CB: **Prediction of mammalian microRNA targets**. *Cell* 2003, **115**(7):787-798.
12. Lewis BP, Burge CB, Bartel DP: **Conserved seed pairing, often flanked by adenosines, indicates that thousands of human genes are microRNA targets**. *Cell* 2005, **120**(1):15-20.
13. Stark A, Brennecke J, Bushati N, Russell RB, Cohen SM: **Animal MicroRNAs confer robustness to gene expression and have a significant impact on 3'UTR evolution**. *Cell* 2005, **123**(6):1133-1146.
14. Brennecke J, Stark A, Russell RB, Cohen SM: **Principles of microRNA-target recognition**. *PLoS biology* 2005, **3**(3):e85.
15. Steinberg D: **MicroRNA Target Practice**. *The Scientist* 2005, **19**(12):14-16.
16. Larranaga P, Calvo B, Santana R, Bielza C, Galdiano J, Inza I, Lozano JA, Armananzas R, Santafe G, Perez A *et al*: **Machine learning in bioinformatics**. *Briefings in bioinformatics* 2006, **7**(1):86-112.
17. Miranda KC, Huynh T, Tay Y, Ang YS, Tam WL, Thomson AM, Lim B, Rigoutsos I: **A pattern-based method for the identification of MicroRNA binding sites and their corresponding heteroduplexes**. *Cell* 2006, **126**(6):1203-1217.
