# Supplementary material for: Structural conservation versus functional divergence of maternally expressed microRNAs in the Dlk1/Gtl2 imprinting region
Source: BMC Genomics. 2008 Jul 23;9:346. doi: 10.1186/1471-2164-9-346 (PMC2500034; doi:10.1186/1471-2164-9-346)
Supplement: Additional file 6 — Gene expression analysis of human targets. [file 1471-2164-9-346-S6.pdf]

Supplementary table S3: Median gene expression and gene expression variance of the human targets.

Tissue	Median gene expression			Gene expression variance		
	Obs. value	z-score	p-value	Obs. value	z-score	p-value
Human Vari targets						
adrenal gland	0.207	4.539	2.824E-06	0.638	-4.543	2.776E-06
amygdala	0.258	4.877	5.376E-07	0.902	-2.357	9.201E-03
bone marrow	0.233	4.875	5.443E-07	0.610	-4.711	1.233E-06
cerebellum	0.257	5.744	4.633E-09	0.713	-2.993	1.380E-03
heart	0.261	5.624	9.336E-09	0.608	-5.489	2.022E-08
hypothalamus	0.260	4.880	5.315E-07	0.834	-3.692	1.113E-04
kidney	0.245	4.992	2.985E-07	0.611	-5.506	1.839E-08
liver	0.220	4.479	3.755E-06	0.667	-5.418	3.005E-08
lung	0.312	5.762	4.151E-09	0.845	-5.185	1.079E-07
lymph node	0.212	4.234	1.147E-05	0.622	-5.081	1.874E-07
ovary	0.185	4.661	1.574E-06	0.571	-3.729	9.631E-05
pancreas	0.209	3.997	3.204E-05	0.573	-5.017	2.626E-07
pituitary	0.191	4.124	1.859E-05	0.558	-4.949	3.724E-07
placenta	0.243	4.323	7.688E-06	0.872	-4.242	1.107E-05
prostate	0.216	4.141	1.728E-05	0.650	-5.329	4.948E-08
salivary gland	0.164	3.644	1.343E-04	0.497	-4.571	2.430E-06
skeletal muscle	0.173	4.765	9.421E-07	0.402	-5.330	4.922E-08
testis	0.198	3.760	8.482E-05	0.557	-5.199	1.001E-07
thymus	0.253	4.434	4.617E-06	0.764	-5.294	5.995E-08
thyroid	0.209	3.570	1.786E-04	0.723	-5.184	1.087E-07
trachea	0.154	3.347	4.091E-04	0.586	-4.986	3.088E-07
uterus	0.165	3.606	1.553E-04	0.505	-4.671	1.498E-06
Conserved human Vari targets						
adrenal gland	0.248	1.634	5.111E-02	0.608	-2.117	1.715E-02
amygdala	0.428	3.157	7.966E-04	0.807	-1.611	5.354E-02
bone marrow	0.162	0.749	2.269E-01	0.510	-2.566	5.142E-03
cerebellum	0.434	4.095	2.114E-05	0.606	-2.128	1.665E-02
heart	0.276	1.960	2.498E-02	0.494	-3.122	8.974E-04
hypothalamus	0.455	3.598	1.600E-04	0.751	-1.996	2.297E-02
kidney	0.326	2.577	4.990E-03	0.545	-2.781	2.713E-03
liver	0.200	1.037	1.499E-01	0.540	-2.988	1.402E-03
lung	0.286	1.438	7.525E-02	0.647	-3.126	8.857E-04
lymph node	0.189	0.924	1.777E-01	0.496	-2.666	3.843E-03
ovary	0.200	1.683	4.617E-02	0.485	-1.901	2.864E-02
pancreas	0.307	2.362	9.081E-03	0.512	-2.233	1.277E-02
pituitary	0.263	2.019	2.176E-02	0.471	-2.537	5.590E-03
placenta	0.343	2.292	1.096E-02	0.815	-1.987	2.347E-02
prostate	0.271	1.704	4.416E-02	0.572	-2.623	4.356E-03
salivary gland	0.184	1.279	1.005E-01	0.419	-2.275	1.146E-02
skeletal muscle	0.173	1.679	4.658E-02	0.373	-2.434	7.465E-03
testis	0.255	1.709	4.375E-02	0.458	-2.964	1.519E-03
thymus	0.255	1.255	1.047E-01	0.583	-3.034	1.205E-03
thyroid	0.249	1.127	1.298E-01	0.589	-2.977	1.454E-03
trachea	0.242	1.793	3.648E-02	0.525	-2.429	7.578E-03
uterus	0.232	1.885	2.973E-02	0.445	-2.216	1.336E-02
