# Supplementary material for: Structural conservation versus functional divergence of maternally expressed microRNAs in the Dlk1/Gtl2 imprinting region
Source: BMC Genomics. 2008 Jul 23;9:346. doi: 10.1186/1471-2164-9-346 (PMC2500034; doi:10.1186/1471-2164-9-346)
Supplement: Additional file 7 — List of imprinted genes in mouse and human used for analysis. [file 1471-2164-9-346-S7.pdf]

Supplementary table S4: Imprinted genes in mouse and human with their corresponding transcript identifiers. These genes were selected for analysis from the Catalogue of Imprinted Genes (http://igc.otago.ac.nz/). 31 human transcripts and 36 murine transcripts were analyzed; transcripts with marginal experimental support were excluded.

**Mouse**		**Human**	
Asb4	NM_023048	ASB4	NM_145872
Atp10a	NM_009728	ATP10A	NM_024490
Cdkn1c	NM_009876	CDKN1C	NM_000076
Commd1	NM_144514	CPA4	NM_016352
Copg2	NM_017478	DIO3	NM_001362
Dlk1	NM_010052	DIRAS3	NM_004675
Gnas	NM_010309	DLK1	NM_001032997
Grb10	NM_010345	DLX5	NM_005221
H19	AK145379	GNAS	NM_000516
Htr2a	NM_172812	GRB10	NM_005311
Igf2	NM_010514	H19	BC053636
Igf2r	NM_010515	HTR2A	NM_000621
Impact	NM_008378	IGF2	NM_000612
Inpp5f	NM_178641	KCNQ1	NM_000218
Kcnq1	NM_008434	L3MBTL	NM_015478
Meg3/Gtl2	NM_144513	MEG3	AF090934
Nap1l5	NM_021432	MEST/PEG1	NM_177524
Ndn	NM_010882	MKRN3	NM_005664
Nnat	NM_010923	NDN	NM_002487
Peg1/Mest	NM_008590	NNAT	NM_005386
Peg10	NM_130877	PEG3	NM_006210
Peg12	NM_013788	PHLDA2	NM_003311
Peg3	NM_008817	PLAGL1	NM_006718
Phlda2	NM_009434	SGCE	NM_003919
Plagl1	NM_009538	SLC22A18	NM_183233
Ppp1r9a	NM_181595	SNRF/SNRPN	NM_022807
Rasgrf1	NM_011245	TP73	NM_005427
Sgce	NM_011360	UBE3A	NM_000462
Slc22a2	NM_013667	WT1	NM_024426
Slc22a3	NM_011395	ZIM2	NM_015363
Slc38a4	NM_027052	ZNF215	NM_013250
Snurf/Snrpn	NM_013670		
Ube3a	NM_011668		
Usp29	NM_021323		
Xlr3b	NM_011727		
Zim1	NM_011769		
