# Supplementary material for: Structural conservation versus functional divergence of maternally expressed microRNAs in the Dlk1/Gtl2 imprinting region
Source: BMC Genomics. 2008 Jul 23;9:346. doi: 10.1186/1471-2164-9-346 (PMC2500034; doi:10.1186/1471-2164-9-346)
Supplement: Additional file 8 — List of eutherian-specific human proteins used for analysis. [file 1471-2164-9-346-S8.pdf]

Supplementary table S5: List of 198 human proteins shared only among eutherians. These proteins were identified using InParanoid Eukaryotic Ortholog Groups (inparanoid.sbc.su.se). For each protein the transcript ID used for target gene predictions as well as the Ensembl protein ID is given.

AB032762	ENSP00000230993	CR607386	ENSP00000329051	NM_018476	ENSP00000361813
AB096939	ENSP00000327696	CR615589	ENSP00000270620	NM_018661	ENSP00000320951
AF078550	ENSP00000371328	DQ227570	ENSP00000330269	NM_018661	ENSP00000320951
AF251510	ENSP00000319204	NM_000586	ENSP00000226730	NM_018690	ENSP00000327669
AF313465	ENSP00000230993	NM_000589	ENSP00000231449	NM_019021	ENSP00000325508
AF329488	ENSP00000357158	NM_000880	ENSP00000263851	NM_020530	ENSP00000215781
AF332961	ENSP00000230993	NM_001001317	ENSP00000307206	NM_020547	ENSP00000257863
AF515828	ENSP00000334042	NM_001001670	ENSP00000341988	NM_020641	ENSP00000369371
AF515829	ENSP00000334042	NM_001001701	ENSP00000296496	NM_021796	ENSP00000352173
AK000959	ENSP00000361780	NM_001004051	ENSP00000339057	NM_021966	ENSP00000216612
AK024084	ENSP00000266542	NM_001004315	ENSP00000372649	NM_024323	ENSP00000254336
AK057129	ENSP00000315870	NM_001008404	ENSP00000333041	NM_024803	ENSP00000369784
AK075085	ENSP00000327669	NM_001011717	ENSP00000366061	NM_030931	ENSP00000371835
AK090409	ENSP00000348635	NM_001012415	ENSP00000298466	NM_031451	ENSP00000253435
AK093122	ENSP00000361556	NM_001012454	ENSP00000367976	NM_031467	ENSP00000230993
AK127066	ENSP00000055335	NM_001013649	ENSP00000304410	NM_032126	ENSP00000323795
AK128106	ENSP00000364734	NM_001013650	ENSP00000328768	NM_032130	ENSP00000257894
AK130550	ENSP00000253362	NM_001014978	ENSP00000364603	NM_032567	ENSP00000369611
AL833894	ENSP00000301165	NM_001017361	ENSP00000359392	NM_032621	ENSP00000361762
AL834344	ENSP00000318131	NM_001024611	ENSP00000341944	NM_032997	ENSP00000363055
AY302593	ENSP00000334805	NM_001037668	ENSP00000347810	NM_033122	ENSP00000273936
AY358362	ENSP00000348635	NM_001037668	ENSP00000347810	NM_033215	ENSP00000055335
AY359075	ENSP00000349709	NM_001891	ENSP00000341030	NM_052938	ENSP00000357158
AY372174	ENSP00000279058	NM_002066	ENSP00000220940	NM_054108	ENSP00000301790
AY471574	ENSP00000334042	NM_002259	ENSP00000352064	NM_054112	ENSP00000253381
AY501002	ENSP00000365486	NM_002287	ENSP00000319204	NM_058203	ENSP00000315764
BC001861	ENSP00000253435	NM_002652	ENSP00000291009	NM_058203	ENSP00000315764
BC001940	ENSP00000362493	NM_003212	ENSP00000296145	NM_080574	ENSP00000253362
BC017947	ENSP00000301165	NM_003309	ENSP00000357597	NM_080753	ENSP00000361726
BC021210	ENSP00000270235	NM_003357	ENSP00000278282	NM_080831	ENSP00000246105
BC025744	ENSP00000340983	NM_003733	ENSP00000257570	NM_130786	ENSP00000263100
BC026183	ENSP00000358957	NM_004223	ENSP00000287156	NM_130794	ENSP00000366208
BC026183	ENSP00000358957	NM_004335	ENSP00000252593	NM_138411	ENSP00000270620
BC028228	ENSP00000321761	NM_004843	ENSP00000263379	NM_138639	ENSP00000246785
BC028368	ENSP00000366191	NM_004942	ENSP00000303532	NM_144602	ENSP00000299191
BC031600	ENSP00000329051	NM_005218	ENSP00000297439	NM_144616	ENSP00000300961
BC032491	ENSP00000287156	NM_005425	ENSP00000325738	NM_144673	ENSP00000268595
BC034222	ENSP00000301790	NM_005480	ENSP00000257909	NM_144692	ENSP00000301165
BC034948	ENSP00000338627	NM_005601	ENSP00000221978	NM_144779	ENSP00000344254
BC035719	ENSP00000263100	NM_005635	ENSP00000366118	NM_145256	ENSP00000340983
BC036772	ENSP00000339057	NM_005699	ENSP00000260049	NM_145275	ENSP00000336871
BC040528	ENSP00000349132	NM_006495	ENSP00000333779	NM_145650	ENSP00000303713
BC040528	ENSP00000349132	NM_006611	ENSP00000023165	NM_145658	ENSP00000312284
BC040528	ENSP00000349132	NM_006664	ENSP00000259631	NM_145762	ENSP00000313423
BC040528	ENSP00000349132	NM_007128	ENSP00000304590	NM_145807	ENSP00000270235
BC040528	ENSP00000349132	NM_007328	ENSP00000352064	NM_152343	ENSP00000331532
BC040528	ENSP00000349132	NM_007333	ENSP00000371328	NM_152539	ENSP00000295622
BC041168	ENSP00000329051	NM_012116	ENSP00000270279	NM_152670	ENSP00000307142
BC041904	ENSP00000364603	NM_013270	ENSP00000326598	NM_152785	ENSP00000309487
BC044215	ENSP00000260049	NM_013439	ENSP00000198536	NM_153233	ENSP00000316130
BC047698	ENSP00000263851	NM_014037	ENSP00000338627	NM_153324	ENSP00000365486
BC051284	ENSP00000315768	NM_014209	ENSP00000368312	NM_153333	ENSP00000361770
BC051688	ENSP00000318131	NM_014330	ENSP00000200453	NM_153757	ENSP00000320488
BC057834	ENSP00000304410	NM_014400	ENSP00000244333	NM_173042	ENSP00000260049
BC062998	ENSP00000336871	NM_014419	ENSP00000221498	NM_173484	ENSP00000361373
BC069285	ENSP00000324895	NM_014450	ENSP00000259608	NM_174918	ENSP00000329920
BC071695	ENSP00000325508	NM_015667	ENSP00000349433	NM_175887	ENSP00000317836
BC073841	ENSP00000336871	NM_015667	ENSP00000349433	NM_177417	ENSP00000336871
BC098247	ENSP00000369784	NM_016546	ENSP00000266542	NM_178494	ENSP00000321761
BC101433	ENSP00000300575	NM_016579	ENSP00000301458	NM_178536	ENSP00000329051
BC101436	ENSP00000300575	NM_016584	ENSP00000228534	NM_181780	ENSP00000333919
BC104004	ENSP00000246785	NM_017559	ENSP00000158009	NM_181788	ENSP00000334805
BC104022	ENSP00000308164	NM_017855	ENSP00000346548	NM_198477	ENSP00000345317
BC107123	ENSP00000368312	NM_018215	ENSP00000318131	NM_198546	ENSP00000364734
BC107124	ENSP00000368312	NM_018410	ENSP00000362493	NM_213726	ENSP00000347129
BC112237	ENSP00000319336	CR607386	ENSP00000329051	X55739	ENSP00000341030
CR602665	ENSP00000319204	CR615589	ENSP00000270620		
